# Synthesis of Non-Racemic Pyrazolines and Pyrazolidines by [3+2] Cycloadditions of Azomethine Imines

**DOI:** 10.3390/molecules23010003

**Published:** 2017-12-21

**Authors:** Franc Požgan, Hamad Al Mamari, Uroš Grošelj, Jurij Svete, Bogdan Štefane

**Affiliations:** 1 Faculty of Chemistry and Chemical Technology, University of Ljubljana, Večna pot 113, SI 1000 Ljubljana, Slovenia; Franc.Pozgan@fkkt.uni-lj.si (F.P.); uros.groselj@fkkt.uni-lj.si (U.G.); jurij.svete@fkkt.uni-lj.si (J.S.); 2 Department of Chemistry, College of Science, Sultan Qaboos University, P.O. Box 36, Al Khoud Muscat, Oman; halmamari@squ.edu.om

**Keywords:** pyrazolines, pyrazolidines, metal-catalyzed cycloadditions, organocatalyzed cycloadditions, azomethine imines, [3+2] cycloadditions

## Abstract

Asymmetric [3+2] cycloadditions of azomethine imines comprise a useful synthetic tool for the construction of pyrazole derivatives with a variable degree of saturation and up to three stereogenic centers. As analogues of pyrrolidines and imidazolidines that are abundant among natural products, pyrazoline and pyrazolidine derivatives represent attractive synthetic targets due to their extensive applications in the chemical and medicinal industries. Following the increased understanding of the mechanistic aspect of metal-catalyzed and organocatalyzed [3+2] cycloadditions of 1,3-dipoles gained over recent years, significant strides have been taken to design and develop new protocols that proceed efficiently under mild synthetic conditions and duly benefit from superior functional group tolerance and selectivity. In this review, we represent the current state of the art in this field and detailed methods for the synthesis of non-racemic pyrazolines and pyrazolidines via [3+2] metal and organocatalyzed transformations reported since the seminal work of Kobayashi et al. and Fu et al. in 2002 and 2003 up to the end of year 2017.

## 1. Introduction

[3+2] Cycloadditions comprise a powerful tool for the construction of five-membered heterocycles [1,2,3,4]. Due to concertedness of the reaction mechanism [4,5,6], [3+2] cycloadditions are also the most straightforward way to obtain a (partially) saturated system with multiple stereogenic centers in a highly stereoselective manner [1,2,3,4,5,6]. The advent of asymmetric catalysis, which also took place in the field of [3+2] cycloaddition chemistry, nowadays enables the preparation of various types of non-racemic saturated heterocycles in a highly efficient manner. Within this context, the use of nitrile oxides, nitrones and azomethine ylides is well established [7,8], whereas asymmetric cycloadditions of azomethine imines remain much less explored [8,9,10,11]. To date, less than three dozen reports on the synthesis of non-racemic pyrazolines and pyrazolidines by [3+2] cycloaddition of azomethine imines can be found in the literature. However, the fact that all these examples have been published since 2003 points at increasing interest in this field.

**Figure 1:**
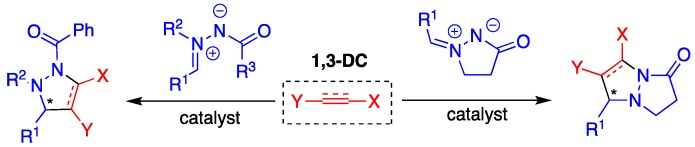
Construction of pyrazoline and pyrazolidine framework via 1,3-dipolar cycloadditions (DCs).

Azomethine imines are 1,3-dipoles of the aza-allyl type represented with iminium amide and diazonium ylide mesomeric structures [9,10,11,12]. They are easily accessible from hydrazine derivatives, diazoalkanes and azo-compounds. Although many azomethine imines are stable compounds, also the unstable dipoles are easily generated in situ from the above precursors. [3+2] Cycloadditions of azomethine imines are of broad substrate scope as the reactions can be performed with all common types of dipolarophiles. Accordingly, reactions with acetylenes provide access to pyrazolines, whereas cycloadditions to olefins are employed to obtain the fully-saturated pyrazolidines. In terms of stereochemistry, [3+2] cycloadditions of azomethine imines furnish products with up to three newly-formed stereogenic centers [9,10,11] (Figure 1). In addition, the pyrazolidine derivatives obtained through these annulations can readily be transformed into differently-protected 1,3-diamine derivatives via N–N bond cleavage with SmI_2_.

**Figure 2:**
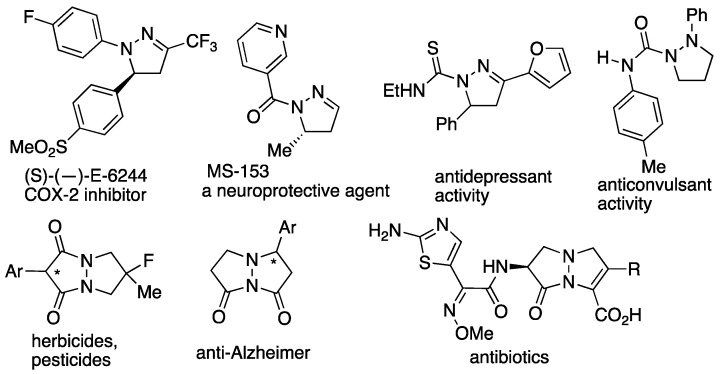
Pyrazolines and pyrazolidines as subunits of bioactive compounds and drugs.

Shintani and Fu have published the first example of asymmetric [3+2] cycloadditions of azomethine imines to acetylenes in 2003. They utilized Cu(I)-based catalyst with chiral phosphaferrocene-oxazoline ligand to obtain the desired cycloadducts regioselectively and in excellent enantioselectivity [13]. Soon after, the same catalyst was also employed in kinetic resolution of azomethine imines by [3+2] cycloaddition [14]. Besides Cu(I)-, also Ag(I)- and Cu(II)-catalysts are amenable in cycloadditions to acetylenes [11,15]. In the reactions of olefins, however, asymmetric organocatalysts comprise the leading approach, although examples of metal-catalysis have also been reported [8,11]. Most probably, the combination of the proven viability of different catalytic systems, as well as the applicability of the pyrazoline scaffold in the development of drugs, agrochemicals, and materials (Figure 2) will continue to spur increased interest in these reactions.

This review covers the synthesis of non-racemic pyrazolines and pyrazolidines by [3+2] cycloadditions of azomethine imines until September 2017. The vast majority of examples are asymmetric catalyzed cycloadditions; however, the preparation of non-racemic cycloadducts by resolution of stereoisomers is also included.

## 2. Synthesis of Pyrazolines by Metal-Catalyzed Cycloadditions

**Scheme 1:**
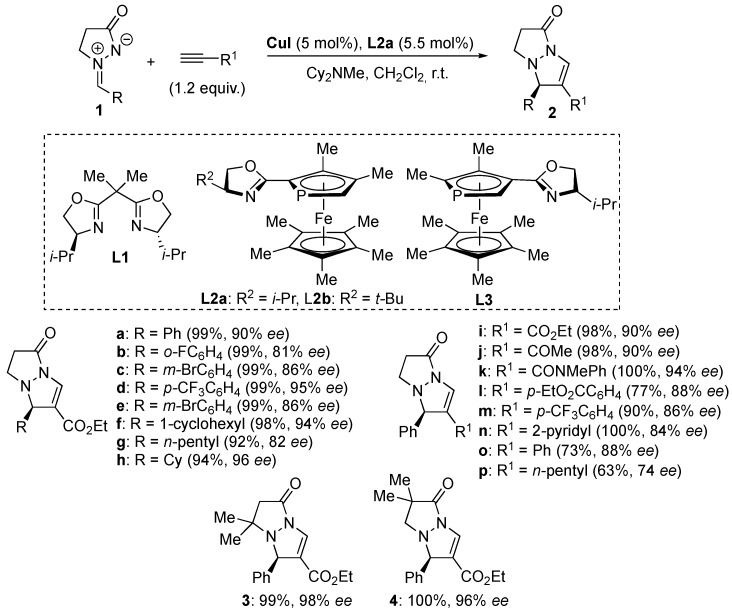
The first Cu(I)-catalyzed asymmetric cycloaddition of azomethine imines with terminal alkynes.

Following the success of copper-catalyzed 1,3-dipolar cycloadditions of alkynes to azides [16,17], and to nitrones [18,19], in 2003, Fu and co-workers extended this useful mode of reactivity to *N*,*N*-cyclic azomethine imines as dipoles. They demonstrated that the reaction of 3-oxopyrazolidin-ium-2-ides **1** with terminal alkynes dramatically accelerated at room temperature in the presence of catalytic amount of CuI, presumably via the formation of copper acetylide as the more reactive dipolarophile, and gave the corresponding pyrazolo[1,2-*a*]pyrazole products **2** as single regioisomers (Scheme 1) [13]. To be able to control the stereoselectivity of the reaction, they utilized enantiomerically-pure oxazolines as privileged chiral ligands, since bidentate phosphines inhibited the catalysis by copper. While the bis(oxazoline) ligand **L1** induced only modest stereoselection in the reaction of azomethine imine **1** (R = Ph) with ethyl propiolate, the level of enantioselectivity was considerably increased in the presence of P,N phosphaferrocene-oxazoline chiral ligand **L2a** bearing an isopropyl substituent on the oxazoline moiety (19% *ee* → 90% *ee*). Introduction of the sterically more demanding *tert*-butyl group (ligand **L2b**) or changing the planar chirality of the phosphaferrocene subunit as in ligand **L3** resulted in a significant decrease of enantiomeric excess (90% *ee* → 58% *ee* and 80% *ee*, respectively). This was the first asymmetric Cu(I)-catalyzed [3+2] cycloaddition of azomethine imines with alkynes.

Their methodology was applicable to azomethine imines derived from aryl-, alkyl- and alkenyl-aldehydes, and thus, they prepared a series of the corresponding enantioenriched pyrazoline derivatives **2** with high chemical yields and *ee* values up to 98%. With regard to the dipolarophile coupling partner, the best yields and *ee* values were obtained with electron-poor alkynes having a carbonyl, an electron-deficient aromatic or heteroaromatic group. Simple aryl- or alkyl-acetylenes also reacted in the presence of the Cu(I)-phosphaferrocene-oxazoline **L2a** catalytic system, although they required gentle heating for a reasonable reaction rate, which consequently resulted in erosion of regioselectivity (~6:1). This protocol tolerates also substituents in the pyrazolidinone ring of an azomethine imine yielding sterically more congested bicyclic products **3** and **4** with excellent yields and enantioselectivities.

**Scheme 2:**
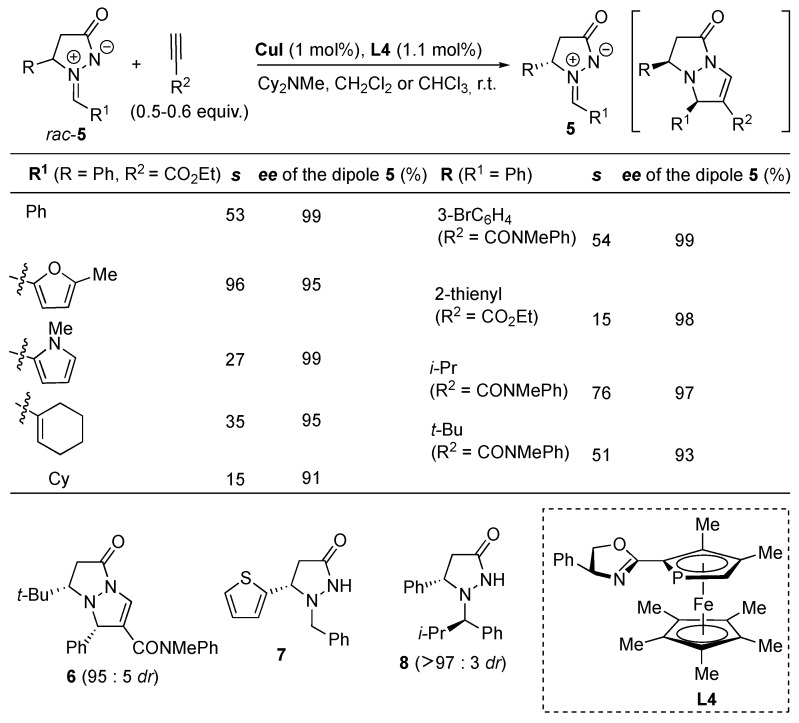
Kinetic resolution of racemic azomethine imines via asymmetric 1,3-dipolar cycloaddition.

Later on, the same research group extended the above-mentioned asymmetric Cu(I)-catalyzed cycloaddition for the efficient preparation of enantiomerically-enriched C5-substituted azomethine imines **5** through kinetic resolution of their racemic mixtures (Scheme 2) [14]. The catalytic reaction of racemic azomethine imines **5** with ethyl propiolate in the presence of the previously used chiral phosphaferrocene-oxazoline ligand **L2a** was less efficient in kinetic resolution than the reaction performed with the catalytic system comprising CuI and ligand **L4** bearing a bigger phenyl group on the oxazoline moiety. The employment of ligand **L4** furnished a two-times higher selectivity factor *s* (*s* = rate of fast-reacting enantiomer/rate of slow-reacting enantiomer) than of ligand **L2a** (49 vs. 27) and allowed the use of even lower catalyst loading (1 mol % vs. 5 mol %). While C4-substituted (e.g., R = cyclohexyl) azomethine imine provided very low selectivity (*s* < 2) in kinetic resolution, a variety of C5-substituted dipoles can be effectively resolved by Cu/**L4**-catalyzed cycloadditions with ester- or amide-substituted alkyne. With this new catalyst system, they succeeded in preparing highly enanatio-enriched azomethine imines (*ee*’s up to 99%), which have a benzylidene, a heteroaryl methylidene, an alkenylidene or a cycloalkylidene substituent at the N1 of the dipole **5**. Enantiomerically-pure azomethine imines **5** prepared in these kinetic resolutions served as precursors for the synthesis of useful pyrazolidinone derivatives **6**, **7** and **8**.

**Scheme 3:**
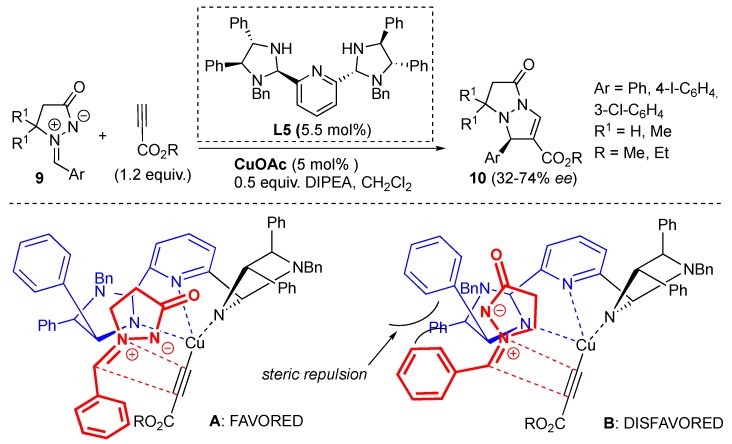
Chiral Py-Bidine ligand in CuOAc-catalyzed cycloaddition of azomethine imines with alkyl propiolates.

Arai et al. developed a chiral (*S*,*S*,*S*,*S*)-bis(imidazolidine)pyridine ligand **L5** (Py-Bidine) and used it in the catalytic asymmetric cycloaddition of cyclic aryl-azomethine imines **9** with alkyl propiolates (Scheme 3) [20]. The best catalytic system, in terms of activity and enantioselectivity, turned out to be the combination of **L5** and CuOAc as a source of Cu^+^ ions, which provided quantitative yield and 60% *ee* of the corresponding (*R*)-cycloadduct **10** (Ar = Ph, R = Et, R^1^ = H) in the reaction of 2-benzylidene-5-oxopyrazolidin-2-ium-1-ide with ethyl propiolate at room temperature. Lowering the reaction temperature (r.t. → –20 °C) and the addition of base *N*,*N*-diisopropylethylamine (DIPEA) significantly improved enantioselectivity in CH_2_Cl_2_ (60% *ee* → 74% *ee*). Examination of different solvents, from nonpolar to polar protic and aprotic (PhMe, EtOH, THF, 1,4-dioxane, MeCN, CH_2_Cl_2_), revealed only a slight solvent effect on the reaction with the exception of MeCN, in which a low chemical yield of the product was obtained. Interestingly, while the use of Cu(I) salts is believed to be crucial for the formation of copper acetylide as a reactive dipolarophile, Cu(II) salts also exhibited significant activity in the presence of ligand **L5**. The authors also proposed a mode of action of the catalyst Cu(I)-**L5** responsible for assuring an asymmetric [3+2] cycloaddition reaction. The in situ generated copper acetylide first coordinates to the (*S*,*S*,*S*,*S*)-Py-Bidine ligand to which (*Z*)-azomethine imine **9** approaches from an upper-side. Because the enantio-faces of the azomethine imine are differentiated on the molecular surface of the (*S*,*S*,*S*,*S*)-(Py-Bidine)–Cu complex, a suitable combination of catalyst and substrate is necessary for a high efficiency. Consequently, the azomethine imine **9** approaches in such a way as to minimize the non-bonded repulsion between the phenyl ring of the Py-Bidine and the aromatic ring of the azomethine imine (model **A**) resulting in bicyclic product **10** with *R* configuration. On the other hand, as presented in model **B**, steric repulsion between phenyl groups prevents the formation of the opposite (*S*)-enantiomer. Although the enantiomeric excesses of the bicyclic pyrazolo[1,2-*a*]pyrazolone products **10** were moderate (32–74%), this newly-designed ligand **L5** has high potential for further development of chiral ligands to promote asymmetric [3+2] cycloaddition reactions. 

**Scheme 4:**
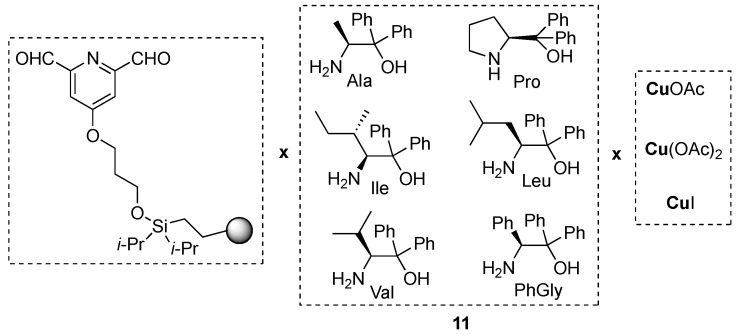
Preparation of chiral polymer-supported Py-Bodine–Cu catalysts.

To further explore the versatility of tridentate chiral pyridine ligands in asymmetric catalysis, the five-membered imidazolidine rings in **L5** were replaced by differently substituted oxazolidines, which can easily be constructed in high optical purity from L-amino acid-derived alcohols **11** and symmetric pyridine(dicarboxaldehyde) [21]. The circular dichroism-high throughput screening approach was utilized to find the most efficient catalyst prepared from the in situ generated chiral polymer-supported bis(oxazolidine)pyridine ligands (Py-Bodine) and copper salts (Scheme 4).

**Scheme 5:**
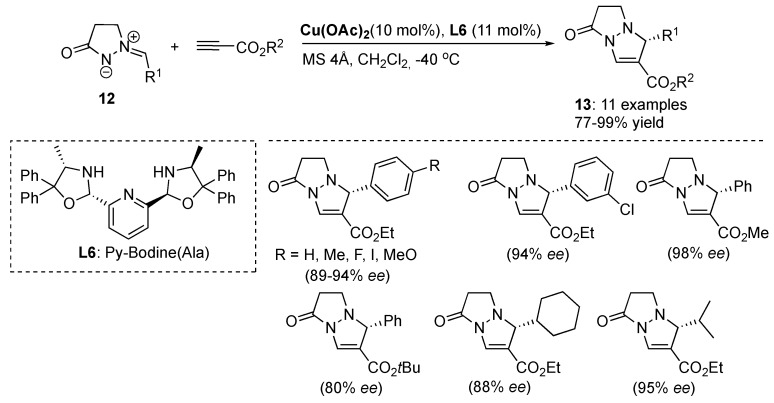
Py-Bodine(Ala)–Cu(OAC)_2_ as a superior catalyst in [3+2] cycloaddition.

Using this custom-made approach, the best candidate in the reaction of azomethine imine **12** (R^1^ = Ph) with ethyl propiolate was established as Py-Bodine(Ala)–Cu(OAc)_2_ furnishing the (*R*)-isomer of the cycloadduct **13** with high chemical yield (99%) and excellent enantioselectivity (94% *ee*). Interestingly, the Py-Bodine ligand prepared from proline-derived alcohol gave the (*S*)-isomer, but with significantly lower enantioselectivity (76% *ee*) for the same reaction. The complex of Py-Bodine(Ala) ligand **L6** and Cu(OAc)_2_ effectively catalyzed the reaction of various azomethine imines **12** bearing aryl or alkyl R^1^ groups with alkyl propiolates providing the corresponding bicyclic pyrazoline products **13** in a highly enantioselective manner (*ee*’s up to 98%) and with nearly quantitative chemical yields in most cases (Scheme 5).

**Scheme 6:**
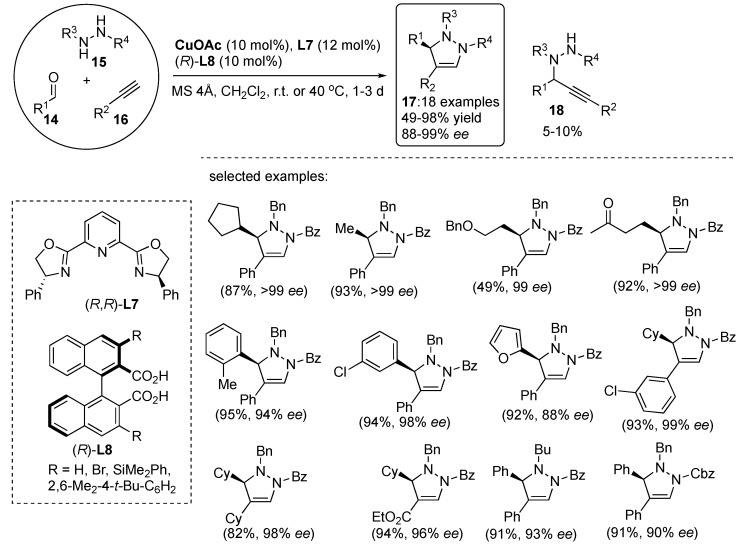
Dual asymmetric induction in three-component reaction for the preparation of enantio-enriched 3,4-disusbtituted pyrazolines

Multicomponent 1,3-dipolar cycloaddition is an attractive strategy for the construction of pyrazolines, particularly where less stable acyclic azomethine imines are used as dipoles. In this context, Marouka et al. reported on the one-pot three-component asymmetric [3+2] cycloaddition reaction of aldehydes **14**, hydrazides **15** and terminal acetylenes **16** by employing the catalytic system composed of CuOAc, pyridine-bisoxazoline (*R*,*R*)-**L7** and binaphthyl dicarboxylic acid (*R*)-**L8** as chiral ligands (Scheme 6) [22]. While the ligand **L7** is proposed to coordinate to the copper center, the chiral Brønsted acid **L8** could interact with the azomethine imine via hydrogen bonding [23]. The labile acyclic azomethine imines generated in situ from aldehyde **14** and hydrazide **15** effectively coupled with the present acetylenes **16** in a highly regioselective and stereoselective manner. The corresponding 3,4-disubstituted pyrazolines **17** formed under mild conditions (r.t. or 40 °C), although for high yields, extended reaction times had to be applied (1–3 days). Notably, the use of axially-chiral dicarboxylic acid as a co-catalyst was not only beneficial to enantioselectivity, but also increased chemoselectivity by suppressing the formation of alkynylation by-product **18**. The most effective acid was found to be binaphthyl dicarboxylic acid (*R*)-**L8** bearing 3,3′-disilyl groups. The methodology was compatible with aliphatic cyclic and acyclic, aromatic and heteroaromatic aldehydes. Moreover, aromatic and aliphatic acetylenes can be used as coupling partners, and various substituents on the hydrazide moiety are tolerated. It was also found that chiral pair matching of ligands **L7** and **L8** is crucial for the efficient asymmetric induction since the combination of (*S*,*S*)-**L7** and (*R*)-**L8** led to a significant drop of the *ee* value (from 99% to 55%) of the pyrazoline product **17** prepared from *N*′-benzylbenzohydrazide, benzaldehyde and phenylacetylene.

**Scheme 7:**
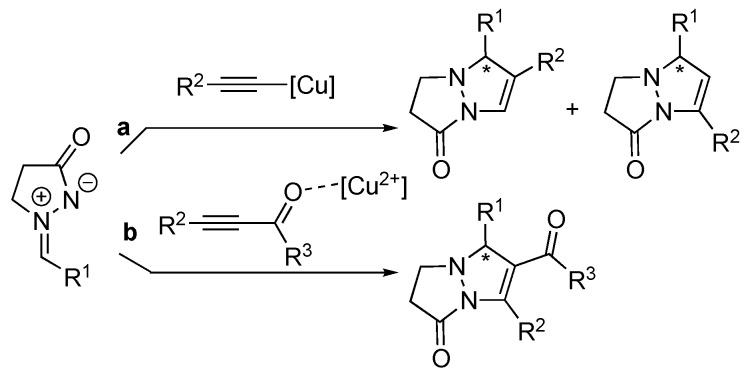
Cu(I)- vs. Lewis acid-catalyzed 1,3-dipolar cycloaddition.

Cu(I)-catalyzed 1,3-cycloaddition of azomethine imines with alkynes is believed to proceed via Cu-acetylide intermediate and is therefore restricted to terminal alkynes (Scheme 7, a). On the other hand, Lewis acid-catalyzed cycloaddition can be applied also to internal alkynes, which importantly extends the use of 1,3-cycloaddition methodology by allowing the synthesis of densely-substituted pyrazoline derivatives (Scheme 7, b).

**Scheme 8:**
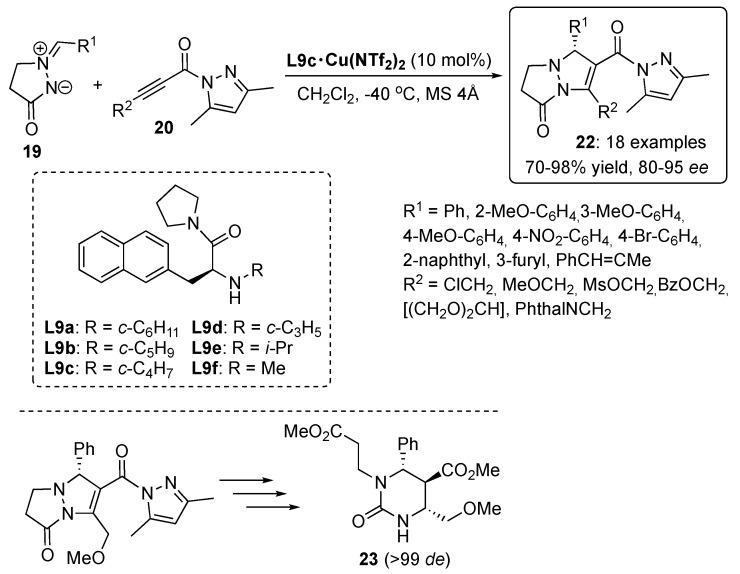
Asymmetric π–cation catalyst-induced cycloaddition of *N*,*N*-cyclic azomethine imines with propioloylpyrazoles.

In this context, the groups of Sakakura and Ishihara reported on the asymmetric [3+2] cycloaddition of *N*,*N*-cyclic azomethine imines **19** with propioloylpyrazoles **20** as dipolarophiles (Scheme 8) [24]. The reaction was catalyzed by the Cu(II)-complex of 3-(2-naphthyl)-L-alanine amide derivative **L9** as chiral ligand, which enabled the preparation of (*R*)-cycloadduct products **22** in good-to-excellent isolated yields (70–98%) and high enantioselectivities (80–95% *ee*). Among Cu(II) salts tested, Cu(NTf_2_)_2_ exhibited the highest reactivity of the chiral catalytic system. This methodology tolerates *meta*- and *para*-substituted phenyl groups, heteroaryl and alkenyl groups on the azomethine imine coupling partner **19**. Besides terminal pyrazole-containing alkyne **20** (R^2^ = H), β-substituted propioloylpyrazoles as disubstituted acetylenes also effectively participated in the present cycloaddition to give fully-substituted pyrazoline derivatives **22**. The activity and enantioselectivity of this novel π–cation catalyst can be finely tuned by changing the *N*-alkyl R group on the ligand **L9**. The synthesized cycloadducts **22** served as optically-active precursors for the synthesis of 1,3-diamines with three contiguous chiral centers, such as in **23**.

**Scheme 9:**
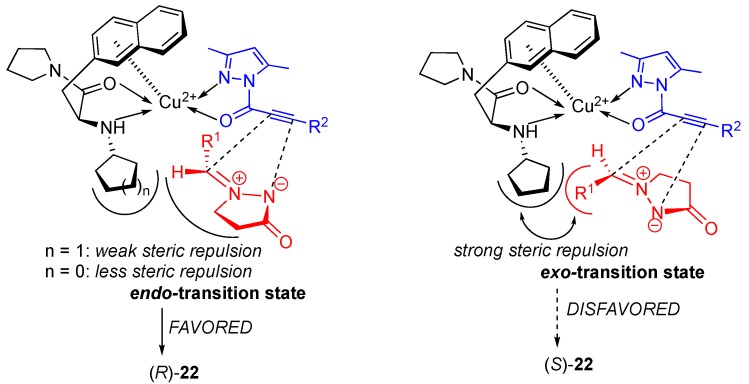
Proposed *endo*- and *exo*-transition states for the asymmetric π–cation catalyst-promoted cycloaddition.

On the basis of experimental observations and previous results [25], a transition state assembly for the asymmetric cycloaddition of **19** and **20** was proposed (Scheme 9). Since the carbonyl *Re* face of a coordinated alkyne is shielded by a naphthyl group through the intramolecular π–cation interaction, an azomethine imine approaches the *Si* face via more favored *endo*-**TS** by avoiding steric repulsion between the R group (e.g., cyclopentyl) of the amide ligand **L9** and the R^1^ group (e.g., Ph) of the azomethine imine **19** to give the corresponding (*R*)-product **22** as the major enantiomer. Less hindered *N*-cycloalkyl groups are successful in avoiding steric repulsion with the ethylene group of **19**. Thus, the most efficient R groups are cyclopropyl and cyclobutyl giving the (*R*)-product **22** (R^1^ = Ph, R^2^ = H) with 93% *ee* and 95% *ee*, respectively, while the use of cyclohexyl and sterically similar isopropyl groups led to a significant decrease of enantioselectivity (54% *ee* and 60% *ee*). The *N*-methyl group, however, was too small for efficient enantio-differentiation. In the *exo*-**TS**, strong steric repulsion between the R^1^ and cyclohexyl groups disfavors the addition of an azomethine imine to a coordinated alkyne, which would otherwise lead to the formation of (*S*)-enantiomeric product **22**.

**Scheme 10:**
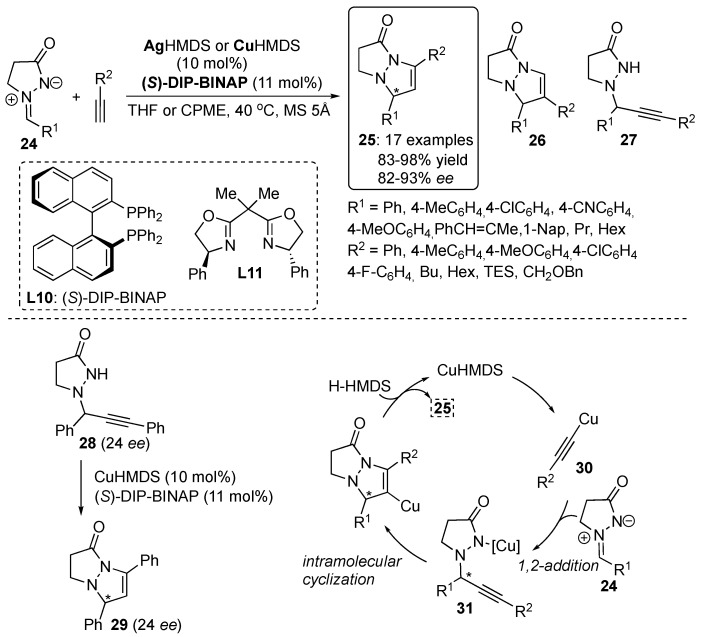
Regioselective formation of 5,7-disubstituted bicyclic pyrazoline derivatives. Tetrahydrofuran = THF, CPME = cyclopentyl methyl ether, MS = molecular sieves, TES = triethylsilyl, DIP-BINAP = 2,2′-bis(3,5-diisopropylphenylphosphino)-1,1′-binaphthalene.

Copper-catalyzed 1,3-dipolar cycloadditions of *N*,*N*-cyclic azomethine imines to terminal alkynes, which proceed via Cu(I)-acetylide intermediate, generally afford 5,6-disubstituted bicyclic products of type **26**. In 2012, Kobayashi et al. developed a silver amide-catalyzed cycloaddition of azomethine imines **24** with terminal alkynes to exclusively afford 5,7-disubstituted bicyclic products **25** (Scheme 10) [26]. This was the first protocol for the synthesis of 5,7-cycloadducts with reversed regioselectivity as in previously reported cycloadditions. It was found out that the strong basicity of AgHMDS was essential for this cyclization reaction. To efficiently suppress the formation of 1,2-adduct **27**, the reaction has to be performed in the presence of molecular sieves using only a slight excess of alkyne. An asymmetric version of this highly regioselective cycloaddition was achieved by utilizing CuHMDS in combination with chiral 2,2′-bis(diphenylphosphino)-1,1′-binaphthalene (BINAP) ligand **L10**. The reaction tolerated electron-deficient and electron-rich aryl, alkenyl and alkyl R^1^ groups on the azomethine imine **24** furnishing the products **25** in high yields and *ee* values in the range of 87–95%. With regard to the terminal alkyne, the reactions proceeded cleanly with electron-deficient and electron-rich aromatic, alkyl, silyl and protected alcohol R^2^ groups, again with high enantioselectivities (*ee*’s up to 93%). Mechanistic studied revealed that, in contrast to the previously proposed concerted cyclization pathway [27], the cycloadduct products **25** are formed via a step-wise reaction mechanism. It involves a 1,2-addition of the metal acetylide **30** to the azomethine imine **24** followed by intramolecular cyclization of the Lewis acid-activated alkyne **31**. The latter assumption was confirmed by successful cyclization of alkyne **28** to bicyclic product **29** with retained *ee* value. While CuHMDS combined with BINAP-type ligand enabled exclusive formation of products **25**, a complete reversal of regioselectivity (formation of **26**) was observed when bisoxazoline **L11** or 2,2′-bipyridyl ligand was used.

## 3. Synthesis of Pyrazolidines

### 3.1. By Metal-Catalyzed Cycloadditions

**Scheme 11:**
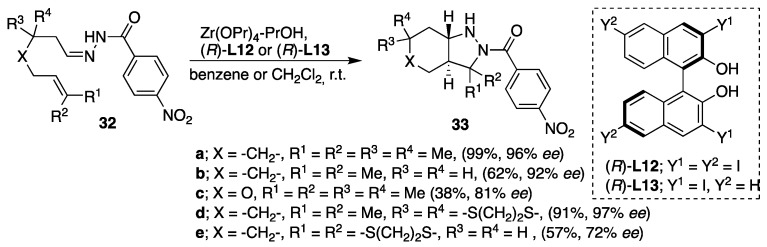
Asymmetric intramolecular [3+2] cycloaddition of hydrazones using a chiral zirconium catalyst.

Acylhydrazones can be visualized as imine equivalents, and their benzoyl derivatives have been shown to react with several nucleophiles under Lewis acid catalytic conditions or in the presence of Lewis base promoter. Acylhydrazones have also been explored as general 1,3-dipoles, and it has been found that the [3+2] cycloaddition reaction of acylhydrazones with olefins is possible under Lewis acid catalytic conditions [28]. Additionally, asymmetric intramolecular [3+2] cycloadditions of acylhydrazones have been described by Kobayashi using chiral zirconium catalyst leading to condensed pyrazolidine derivatives [29]. The chiral zirconium catalyst was prepared in situ from Zr(OPr)_4_ (10 mol %) and selected (*R*)-BINOL ligand (12 mol %). The reaction was run in benzene or dichloromethane in the presence of 50 mol % of PrOH as an additive at room temperature yielding the corresponding fused pyrazolidine derivatives as major *trans* isomers (*cis*/*trans* ratio > 1/99) and with *ee* from 72 up to 97% (Scheme 11). Although that precise reaction pathway in these reactions is not clarified, it was clearly shown that *cis*/*trans* selectivity was controlled by the chirality of the chiral catalyst. For example, when (*S*)-**L12** was used as a catalyst instead of (*R*)-**L12** under the same reaction conditions, the corresponding *cis* isomer of cycloadduct of (*S*)-citronellal-derived 4-nitrobenzoylhydrazone was obtained with good selectivity.

**Scheme 12:**
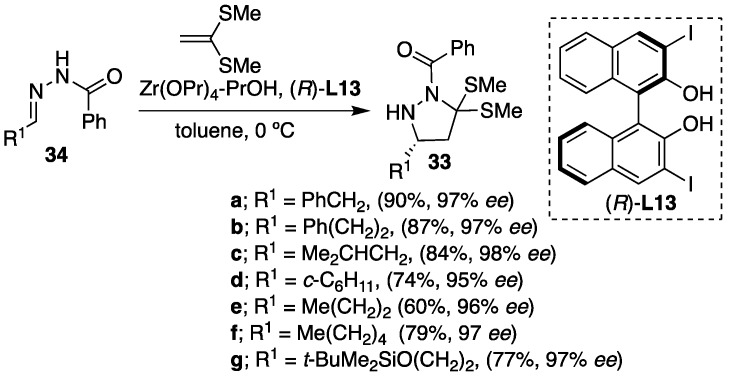
Asymmetric intermolecular [3+2] cycloaddition of hydrazones with ethene-1,1-diylbis(methylsulfane) using a chiral zirconium catalyst.

However, in this methodology, the substrate scope was restricted, and the reactions were limited to only an intramolecular type of [3+2] cyclisation. Later on, the same group developed the efficient zirconium-catalyzed enantioselective [3+2] intermolecular cycloaddition of hydrazones to olefins leading to optically-active pyrazolidine, pyrazoline and 1,3-diamine derivatives [30]. In this case, benzoylhydrazones were found to be superior substrates over the 4-nitrobenzoyl analogues. The amount of olefin used also effected the reactivity, and the yields were significantly improved by using two equiv. of substituted olefins. Hydrazones derived from a β-branched aldehyde, a sterically-hindered aldehyde, enolizable aldehydes and functionalized aldehydes all readily reacted with ethene-1,1-diylbis(methylsulfane) without any side reactions, and the corresponding substituted pyrazolidines **35** were obtained in high yield and high levels of enantioselectivity (Scheme 12).

**Scheme 13:**
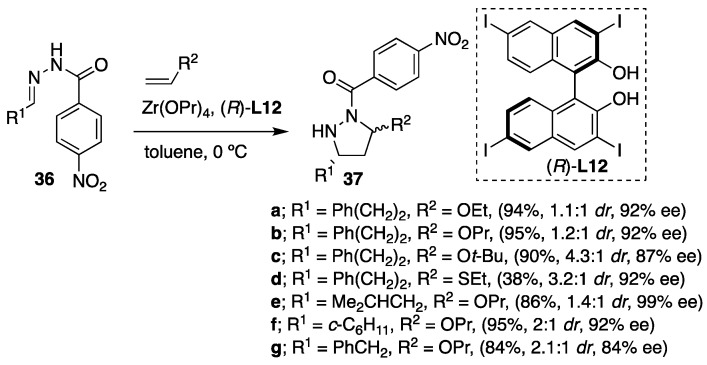
Asymmetric intermolecular [3+2] cycloaddition of hydrazones with ethene-1,1-diylbis(methylsulfane) using a chiral zirconium catalyst.

Furthermore, the variety of hydrazones derived from α-branched and β-branched aliphatic aldehydes reacted readily with different vinyl ethers as olefins furnishing the pyrazolidines **37** containing a *N*,*O*-acetal functionality in high yields and enantioselectivities (Scheme 13). In this particular case, *p*-nitrobenzoyl hydrazones were found to be more suitable derivatives than their benzoyl analogues due to their higher reactivity under the catalytic conditions. However, moderate diastereoselectivities were obtained in the majority of the cases (diastereomer ratio (*dr*) > 1/1), whereas both diastereomers showed excellent enantioselectivity in most instances (*ee* > 81%). Concerning the mechanistic aspect of the transformations, the authors suggested a concerted reaction pathway over the alternative stepwise pathway. The mechanism of the transformation was among the other facts elucidated on the observation that both diastereomers of the products have the same absolute configuration at the carbon atom bearing the R^1^ substituent (Scheme 13).

**Scheme 14:**
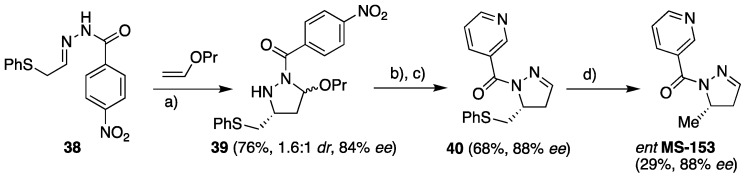
Synthesis of *ent* **MS-153** pyrazoline derivative. (**a**) Zr(OPr)_4_ (20 mol %), (*R*)-3,3’-I_2_BINOL ((*R*)-**L13**) (24 mol %), toluene, 10 °C, 24 h; (**b**) LiAlH_4_, THF, −78 °C; (**c**) nicotinoyl chloride hydrochloride, *i*-Pr_2_EtN, DMAP, r.t.; (**d**) Raney-Ni (W-2), EtOH-acetate buffer, r.t.

The developed methodology was elegantly applied for the synthesis of enantioenriched compound **MS-153** of biological importance. The [3+2] cycloaddition of hydrazone **38** to propyl vinyl ether proceeded with 76% yield and 84% *ee* for the major and 97% *ee* for the minor isomer. The *p*-nitrobenzoyl functionality was removed by treatment with LiAlH_4_ at −78 °C in THF followed by nicotinoylation. The obtained intermediate **40** was a useful starting material for the synthesis of **MS-153** derivatives. However, after the removal of the phenylthio group using Raney-Ni, *ent* **MS-153** was obtained with high enantioselectivity (88% *ee*) (Scheme 14).

**Scheme 15:**
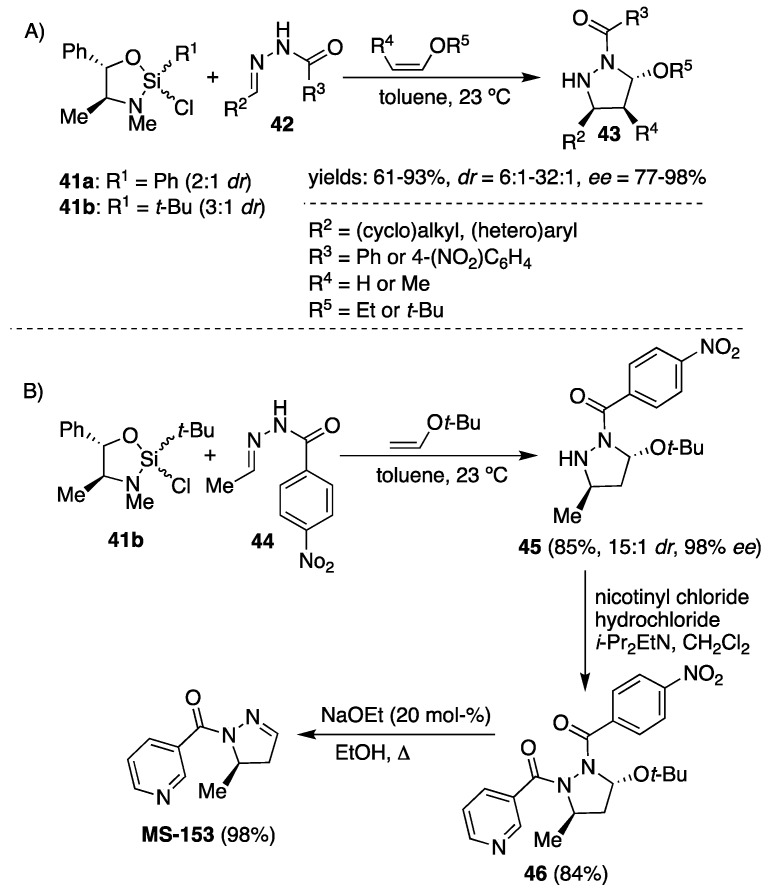
(**A**) Chiral silicon Lewis acid mediated asymmetric synthesis of 3,4,5-substituted pyrazolidines; (**B**) application of the methodology for the enantioselective synthesis of (*R*)-**MS-153**.

Phenyl and *tert*-butylsilane reagents (easily prepared from pseudoephedrine and phenyl or *tert*-butyltrichlorosilane as a mixture of diastereomers at silicon) introduced by Leigghton et al. were applied for acylhydrazone-enol ether cycloadditions [31]. Phenylsilane was found to facilitate the cycloaddition of *N*′-(3-phenylpropylidene)benzohydrazide with ethoxyethene to yield the corresponding pyrazolidine in 61% with 6:1 diastereoselectivity and 77% *ee*. The use of *tert*-butyl vinyl ether resulted in significant improvement in both diastereoselectivity (*dr* 24:1) and enantioselectivity (90% *ee*), and the transformation was found to perform the best in toluene at room temperature (Scheme 15). Variety of benzoylhydrazones derived from aliphatic (Ph(CH_2_)_2_CHO, BnOCH_2_CHO, CyCHO, *t*-BuCHO) and (hetero)aromatic (benzaldehyde, 4-fluorobenzaldehide and furan-2-carbaldehyde) aldehydes reacted readily with *tert*-butyl vinyl ether (three equiv.) in the presence of phenylsilane (1.5 equiv.), yielding 3,5-disubstituted pyrazolidines **43** up to 85% yield, >19:1 *dr* and *ee*’s above 90% (Scheme 15). Unlike the Zr(II)-catalyzed cycloaddition of benzoylhydrazones **36** (Scheme 13), in this case, the β-substituted enol ethers reacted highly stereoselectively (>10:1 *dr*, 97% *ee*) with *N*′-(3-phenylpropylidene)benzohydrazides **42** providing enantio-enriched 3,4,5-trisubstituted pyrazolidine **43** (Scheme 15, Example A). Selectivity with the acetaldehyde-derived hydrazone (R^2^ = Me, Scheme 15) is significantly reduced relative to that observed with larger R^2^ groups when phenylsilane **41a** is applied. Replacing the phenyl moiety in chiral silicon Lewis acid **41a** with sterically more demanding *tert*-butyl group **41b** resulted in significant improvement in stereoselectivity when more electrophilic *N*′-ethylidene-4-nitrobenzohydrazide **44** was reacted with *tert*-butyl vinyl ether to provide (5*R*)-3-(*tert*-butoxy)-5-methylpyrazolidine **45** (Scheme 15, Example B) in good yield of 85% and stereoselectivity (>15:1 *dr*, 98% *ee*). Two additional synthetic steps furnished the targeted **MS-153** substrate in 70% overall yield and > 99% *ee* (Scheme 15, Example B) [32]. Furthermore, a concise synthesis of manzacidin C based on the second generation [33] chiral silicon Lewis acid, which promoted diastereo- and enantioselective acylhydrazone-alkene [3+2] cycloaddition, has also been reported by the same group [34]. The corresponding enantioenriched 3,5,5-trisubstituded pyrazolidine precursor was subsequently used for the synthesis of crucial 1,3-bisamide intermediate, which was obtained in 73% yield and high stereoselectivity (>20:1 *dr* and 94% *ee*).

**Scheme 16:**
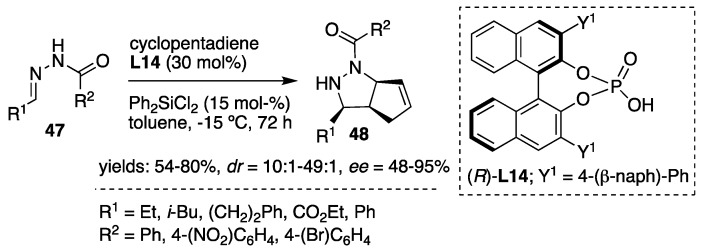
1,3-Dipolar cycloaddition of acylhydrazones with cyclopentadiene catalyzed by cooperative action of chiral Lewis and Brønsted acids.

In addition to the above-described silicon Lewis acid-promoted [3+2] cycloadditions of acylhydrazones, Tsogoeva et al. [35] developed a catalytic system comprised of chiral Brønsted acid (chiral BINOL phosphates) and silicon Lewis acid (R_2_SiX_2_). The combination of both acids acted cooperatively in the stereoselective intermolecular cycloaddition of acylhydrazones with cyclopentadiene providing a convenient and facile process for the synthesis of pyrazolidines. Assumed synergism between the BINOL phosphate (*R*)-**L14** and Ph_2_SiCl_2_ was also tested. Lewis acid (dichlorodiphenylsilane) alone was inactive in catalysis, whereas BINOL phosphate itself showed low reactivity and moderate enantioselectivity (99:1 *dr*, 47% *ee*, example: R^1^ = Et, R^2^ = 4-(NO_2_)-C_6_H_4_, Scheme 16). The combination of both components in ratio (*R*)-**L14**/ Ph_2_SiCl_2_ in 2/1 was established to be the most active, producing the corresponding pyrazolidines **48** as major *cis* isomers in good to excellent enantioselectivities (Scheme 16).

**Scheme 17:**
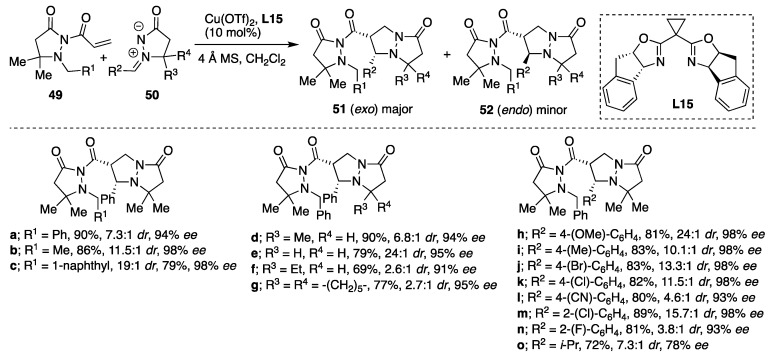
Copper(II)-catalyzed enantioselective [3+2] cycloadditions of *N*,*N*′-cyclic azomethine imines with 2-acryloyl-3-pyrazolidinones.

Copper(II)-catalyzed diastereo- and enantioselective cycloadditions of azomethine imines to 2-acryloyl-3-pyrazolidinones were developed by Sibi et al. deriving the corresponding *exo* cycloadducts with high diastereoselectivities (up to > 96:4 *cis*/*trans*) and enantioselectivities for *cis* isomer up to 98% *ee* [36]. The Cu(OTf)_2_/**L15**-catalytic system was found to be the most efficient in producing the corresponding cycloadducts **51** as a major *syn* diastereomers and with excellent enantioselectivities (Scheme 17). Chiral Lewis acid prepared from Cu(OTf)_2_ and additional bis(oxazoline) ligands such as *t*-Bu-BOX, Ph-BOX and Bn-BOX were also studied as catalysts; however, when the Cu(OTF)_2_/*t*-Bu-BOX system was used as a catalyst, *syn* products were formed in good yields and with lower diastereoselectivity. On the other hand, Cu(OTF)_2_/Ph-BOX and Cu(OTF)_2_/Bn-BOX catalytic systems behaved similarly yielding *exo*/*endo* product in ~5/1 and 82% *ee* of the major isomer. When applying the Cu(OTf)_2_/**L15**-catalytic system, generally excellent yields and good diastereo- and high enantioselectivities were possible from cycloadditions of a variety of C5 and *N*′-arylmethylidene-substituted azomethine imines **50** and different α,β-unsaturated pyrazolidinone imides **49** (Scheme 17). However, when the methodology was extended to β-substituted α,β-unsaturated pyrazolidinone imides such as pyrazolidinone chrotonate, no reaction was observed or the cycloadducts were formed in moderate yields and in low enantioselectivities.

**Scheme 18:**
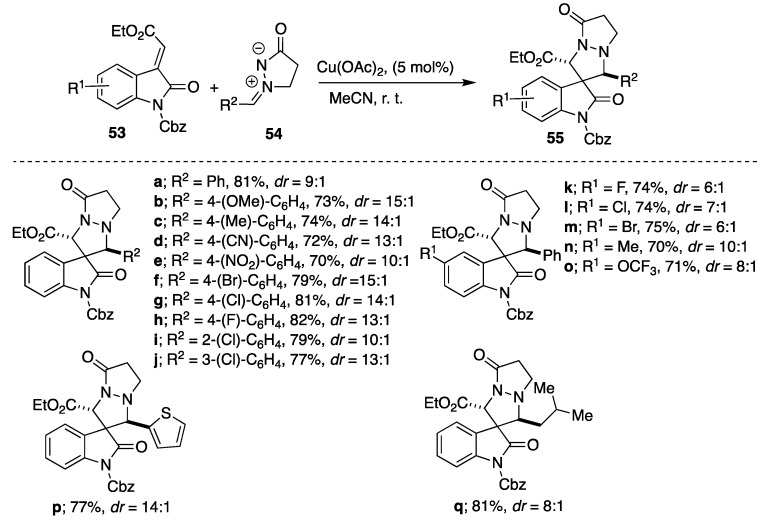
Cu(AcO)_2_-catalyzed diastereoselective [3+2] cycloadditions of *N*,*N*′-cyclic azomethine imines with methyleneindolinones.

An additional example of copper(II)-catalyzed [3+2] cycloadditions of *N*,*N*′-cyclic azomethine imines is Cu(OAc)_2_-catalyzed cycloaddition of azomethine imines to methyleneindolinones described by Sun and coworkers [37]. Among tested Lewis acids (Cu(OAc)_2_, AgOTf, Sc(OTf)_3_, Zn(OTf)_2_, CuI, Cu(acac)_2_ and Cu[MeCN]_4_PF_6_), Cu(OAc)_2_ was found to be the most efficient in acetonitrile as a solvent of choice. Generally, when Cu(OAc)_2_ was used as a Lewis acid catalyst, the reactions proceeded smoothly to afford the desired spiro adducts **55** in moderate to high yields (70–82%) and good stereoselectivities (9:1 up to 15:1) forming *trans* diastereomer as the major isomer (Scheme 18). Notably, the heteroaryl (2-thienyl) and alkyl substituted azomethine imines were tolerated in the reaction and derived the corresponding products **55p** and **55q** in 77%, 14:1 *dr* and 81%, 8:1 *dr*, respectively (Scheme 18). An enantioselective variant of this reactions was also carried out screening a series of chiral ligands (*i*-Pr- and *t*-Bu-substituted oxazoline ligands and *i*-Pr-Phosferrox ligand), affording the corresponding product **55a** in moderate yield and low enantioselectivity (up to 48% *ee*).

**Scheme 19:**
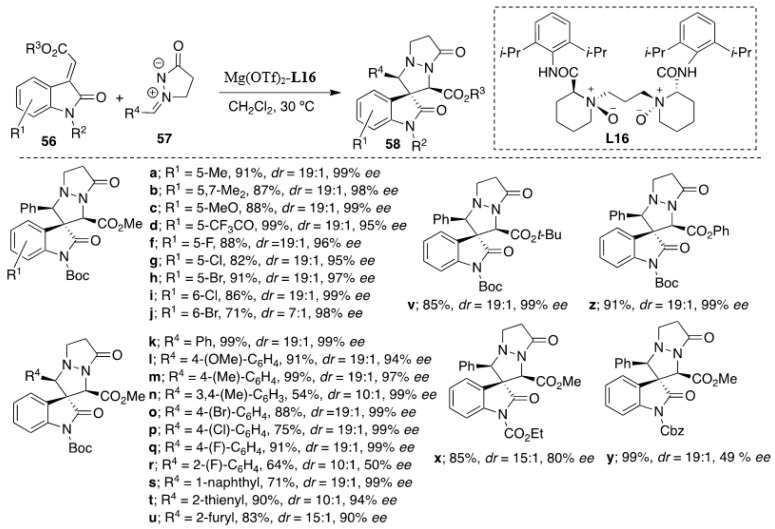
Stereoselective *N*,*N*′-dioxide-Mg(OTf)_2_-catalyzed 1,3-dipolar cycloaddition of methyleneindolinones with *N*,*N*′-cyclic azomethine imines.

Recently, Feng et al. developed highly diastereo- and enantioselective [3+2] cycloaddition of methyleneindolinones with *N*,*N*′-cyclic azomethine imines, which was successfully catalyzed by a *N*,*N*′-dioxide-ligated-Mg(OTf)_2_ complex [38]. It should be stressed that unlike in the above-described Cu(OAc)_2_-catalyzed examples [37], in this case, the major *cis* diastereomer prevailed. Three different alkaline metal salts (Ca(OTf)_2_, Ba(ClO_4_)_2_, and Mg(OTf)_2_ were tested in combination with several chiral *N*,*N*′-dioxide ligands derived from (*S*)-proline, (*S*)-Ramipril and (*S*)-pipecolic acid. However, the complex of Mg(OTf)_2_ could offer only one isomer in nearly quantitative yield in the test reaction. The optimized reaction conditions constituting of 5 mol % of catalyst (**L16**-Mg(OTf)_2_, 1:1) in dichloromethane at 30 °C in 48 h yielded the corresponding spiro adducts in excellent yields and enantioselectivities (Scheme 19). Methyleneindolinones bearing substituents at different positions of the phenyl ring had little influence on the selectivity (*dr* 19:1 in most cases with *ee*’s 90–99%). Furthermore, good results were also obtained when changing the electronic and steric nature at *meta*- or *para*-position on the aryl moiety of the *N*,*N*′-cyclic azomethine imines. However, the azomethine imines with a substituent at the *ortho* position in the phenyl ring in general gave lower yields of cycloadducts with decreased enantioselectivity (e.g., R^2^ = 2-F-C_6_H_4_, 64% yield, 50% *ee*, Scheme 19). The relationship between the enantiomeric excess of the cycloadduct and **L16** showed a weak negative nonlinear effect, suggesting that both monomeric and oligomeric catalytic species exist in the solution, but the monomeric complex might act as the more active one since its existence was clearly confirmed by observing the molecular mass of [**L16** + Mg^2+^ + OTf^−^] in ESI-MS spectra.

**Scheme 20:**
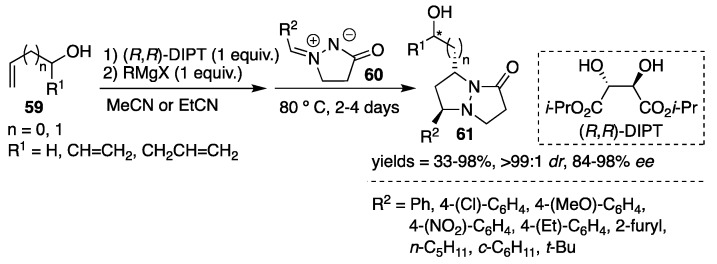
The stereoselective 1,3-dipolar cycloaddition of *N*,*N*′-cyclic azomethine imines with allylic alcohols by the use of Grignard reagents and diisopropyl (*R*,*R*)-tartrate.

An attractive and unique 1,3-dipolar cycloaddition reaction of azomethine imines **60** to allylic [39,40] and homoallylic [41] alcohols **59**, based on a magnesium-mediated multinucleating chiral reaction system utilizing diisopropyl (*R*,*R*)-tartrate [(*R*,*R*)-DIPT] as a chiral reagent was developed by Ukaji and Inomata et al. The transformation was shown to be applicable to both aryl- and alkyl-substituted azomethine imines. The corresponding optically-enriched *trans*-pyrazolidines **61** were obtained with excellent regio-, diastereo- and enantioselectivity, with results up to 98% *ee* (Scheme 20). As an example, a mixture of allyl alcohol and (*R*,*R*)-DIPT was reacted with alkylmagnesium halide as a magnesium source followed by the addition of azomethine imine. The reactions were performed at 80 °C and alkanonitriles (MeCN or EtCN) were found to be the most suitable solvents. The use of a catalytic amount of (*R*,*R*)-DIPT was also effective when accompanied by the addition of one equiv. of MgBr_2_. The proposed transition state model consists of a carbonyl oxygen of azomethine imine, rather than imine nitrogen, coordinated to the magnesium salt of (*R*,*R*)-DIPT. In this model, the *pro*-*R* allyloxy moiety approaches the azomethine imine group, giving the *R*,*R*,*R*-configuration (confirmed by the X-ray analysis of cycloadduct derivatives) of the cycloadducts.

**Scheme 21:**
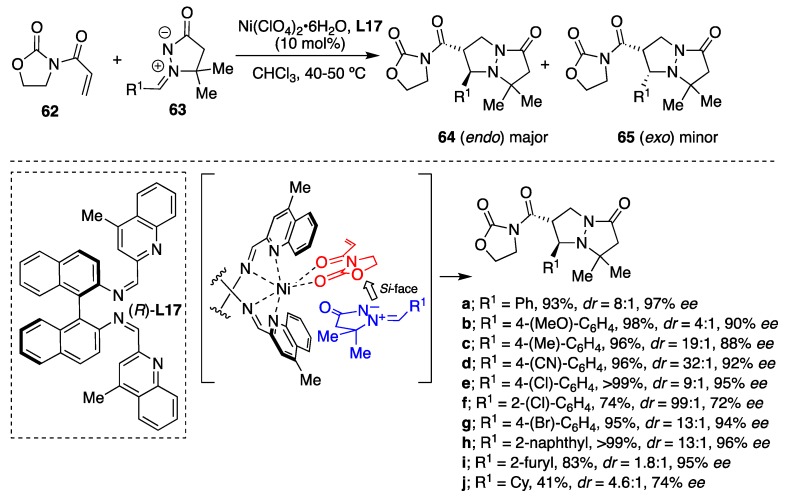
(*R*)-BINIM-4Me-2QN–Ni(II)-catalyzed asymmetric [3+2] cycloadditions of *N*,*N*′-cyclic azomethine imines with 3-acryloyl-2-oxazolidinone.

The diastereodivergent process leading to enantiopure pyrazolidine derivatives **64** and **65** from 3-acryloyl-2-oxazolidinone **62** and *N*,*N*′-cyclic azomethine imines **63** employing Ni(II)-catalyzed asymmetric [3+2] cycloadditions was developed [42]. Among the tested catalytic systems, the in situ formed binaphthyl-quinolinediimine-based Ni(II) complex, (*R*)-**L17**–Ni(II), exhibited favorable activity yielding the corresponding pyrazolidine derivatives **64** as a major *trans* diastereomer and with high enantioselectivity. Under the catalysis of (*R*)-**L17**–Ni(ClO_4_)_2_·6H_2_O in chloroform at 40–50 °C and in the presence of molecular sieves (MS) 4 Å, the reaction proceeded well and was independent of the electronic character of the benzene ring substituents (Scheme 21). It is of note here that the enantio- and diastereoselectivity improved in some cases upon increasing the reaction temperature from room temperature up to 50 °C, which could be attributed to enhanced ligand exchange rate of the transition state Ni(II)-complex. Although the reaction of C-cyclohexyl substituted azomethine imine resulted in moderate yield and 74% *ee*, the cycloaddition of naphthyl and heteroaryl analogues afforded excellent enantioselectivities (Scheme 21).

The results of the Ni(II)-catalyzed reaction are comparable to those of analogous Cu(II)-catalyzed reaction [36], where *exo*-cycloadduct (yielding the *cis*-diastereomer) was the major product, complementarily since in these cases, the *endo*-cycloaddition occurred. The difference in diastereoselectivity between the Cu(II)- and the Ni(II)-catalyzed cycloaddition of structurally-similar substrates can be explained by the alternative approach of the azomethine imine dipole depending on the metal geometry of catalytic chiral Lewis acid complex. The Cu(II)-catalyzed cycloaddition most probably proceeds via a distorted square-planar complex, which favors the *exo*-approach of a dipole. However, the Ni(II)-catalyzed [3+2] cycloaddition likely proceeds through an octahedral complex forcing the *endo*-approach of a dipole.

**Scheme 22:**
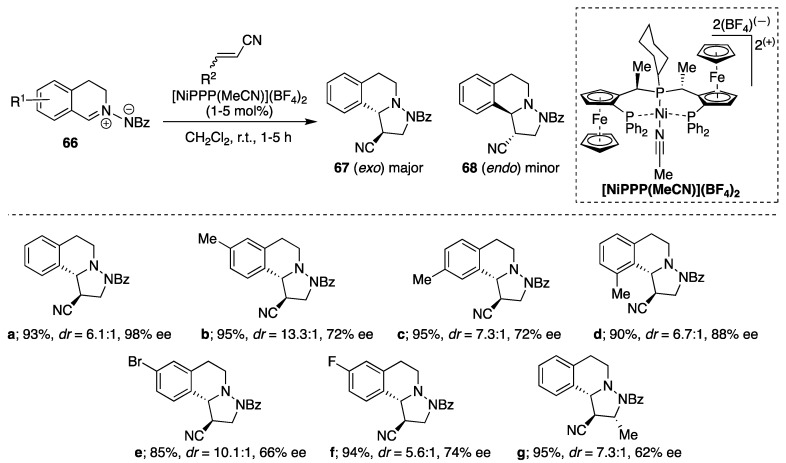
Ni(II)-Pigiphos-catalyzed 1,3-dipolar cycloaddition of functionalized *C*,*N*-cyclic azomethine imines to cyanoolefins.

Togni et al. developed Ni(II)-Pigiphos enantioselective 1,3-dipolar cycloaddition of various *C*,*N*-cyclic azomethine imines **66** to α,β-unsaturated nitriles [43]. When *N*-benzoylamino-3,4-dihydroisoquinolinium betaine was reacted with acrylonitrile in the presence of 5 mol % of [Ni(PPP)(MeCN)](BF_4_)_2_ in dichloromethane, the completion of the reaction was observed after 0.5 h yielding 3,4-regioisomer, with not only good diastereomeric access of the *trans*-isomer, but also excellent enantioselectivity (96% *ee*). Under the optimized reaction conditions, the substrates functionalized with different electron-donating or electron-withdrawing groups yielded moderate enantiomeric excesses, form 60% up to 88% *ee* (Scheme 22). The *exo*-selectivities were good to moderate, decreasing from 13.3:1 to 5.6:1 in decreasing the electron-donating ability of the substituent (Me, H, Br, F) on the position para to the 1,3-dipole. The relative *trans*-stereoselectivity of the major cycloadduct **67** was established in each case by ^1^H and 2D NMR spectroscopy. Furthermore, the absolute configurations (3*R*,4*R*) could be established by X-ray crystallography of enantiopure single crystals of the major diastereomer. The potential influence of a more functionalized cyanoolefins on the *exo*-selectivity of the transformation was also investigated using crotononitrile, methacrylonitrile, *trans*-cinnamonitrile and *cis*-2-pentenonitrile. The reactivity of this substituted acrylonitriles was much more inferior compared to the acrylonitrile, as no cycloaddition occurred with *trans*-cinnamonitrile and *cis*-2-pentenenitrile under the same reaction conditions. Furthermore, methacrylonitrile yielded 52% of the corresponding regioisomeric mixture of cycloadducts (3,4-cycloadduct/3,5-cycloadduct, 3.7/1) after 48 h at 40 °C. However, addition of crotononitrile (four equiv., *cis*/*trans* mixture) to *N*-benzoylamino-3,4-dihydroisoquinolinium betaine at r.t. over two days resulted largely in the formation of the *trans*-3,4-cycloadduct (*dr* = 7.3/1) in 66% yield and 62% *ee* of the *trans*-isomer (Scheme 22, e.g., **67g**). On the basis of the above-mentioned results, it can be concluded that the steric size of the dipolarophile is a crucial factor in these 1,3-dipolar cycloadditions.

**Scheme 23:**
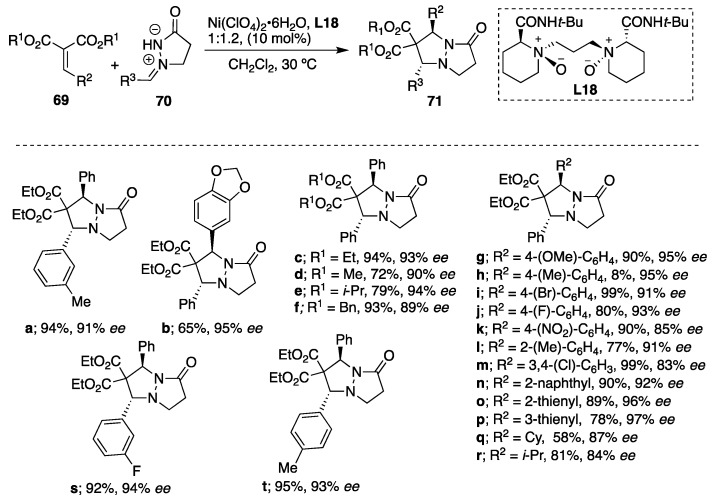
Enantioselective *N*,*N*′-dioxide-Ni(ClO_4_)_2_·6H_2_O-catalyzed 1,3-dipolar cycloaddition of alkylidene malonates with *N*,*N*′-cyclic azomethine imines.

Feng et al. represented the first example of asymmetric reaction of azomethine imines **70** with alkylidene malonates **69** using *N*,*N*′-dioxide-Ni(II) complexes [44]. Different metal sources such as Sc(OTf)_3_, Mg(ClO_4_)_2_ and Co(ClO_4_)_2_·6H_2_O were evaluated; however, the *N*,*N*′-dioxide-Ni(ClO_4_)_2_·6H_2_O promoted the reaction giving rise to the corresponding endo-cycloadduct **71** as a single isomer (*dr* > 99:1). Different nickel sources were also examined (Ni(BF_4_)_2_·6H_2_O and NiBr_2_), but no improvement in the cycloaddition was observed. The counter anion has a crucial effect on both the yield and the selectivity in the azomethine imine-alkylidene malonate cycloaddition. Upon the optimization of the reaction conditions, the ratio of 1:1.2 between Ni(ClO_4_)_2_·6H_2_O and *N*,*N*′-dioxide ligand **L18** was chosen as optimal, and the reaction performed the best in dichloromethane. It is important to note that the solvent effects the reaction greatly since very low conversions were obtained in solvents such as toluene, THF, MeCN and EtOAc. The catalytic composition was also investigated, and a slightly positive nonlinear effect was obtained between the enantiomeric excess of the ligand and the product, suggesting that the minor oligomeric aggregation of the *N*,*N*′-dioxide-Ni(ClO_4_)_2_·6H_2_O might exist in the catalytic system. However, the ESI-MS studies of Ni(ClO_4_)_2_·6H_2_O and chiral ligand in a ratio of 1:1.2 in dichloromethane confirmed the existence of the monomeric nickel species [**L18** + Ni^(2+)^ + ClO_4_^−^] in solution. Using the optimized catalytic system, a variety of dipolarophiles, (cyclo)alkyl- and (hetero)aryl-substituted alkylidene malonates, reacted with *N*,*N*′-cyclic azomethine imines, deriving the corresponding products as sole *trans*-diastereomers with excellent enantioselectivities (from 83% up to 97% *ee*) (Scheme 23). To demonstrate the scalability, a gram-scale reaction was performed between diethyl 2-benzylidenemalonate and (*Z*)-1-benzylidene-1λ^4^-pyrazolidin-3-one under the optimized catalytic system to give the corresponding cycloadduct in 85% yield and 92% *ee*.

### 3.2. By Organocatalyzed Cycloadditions

**Scheme 24:**
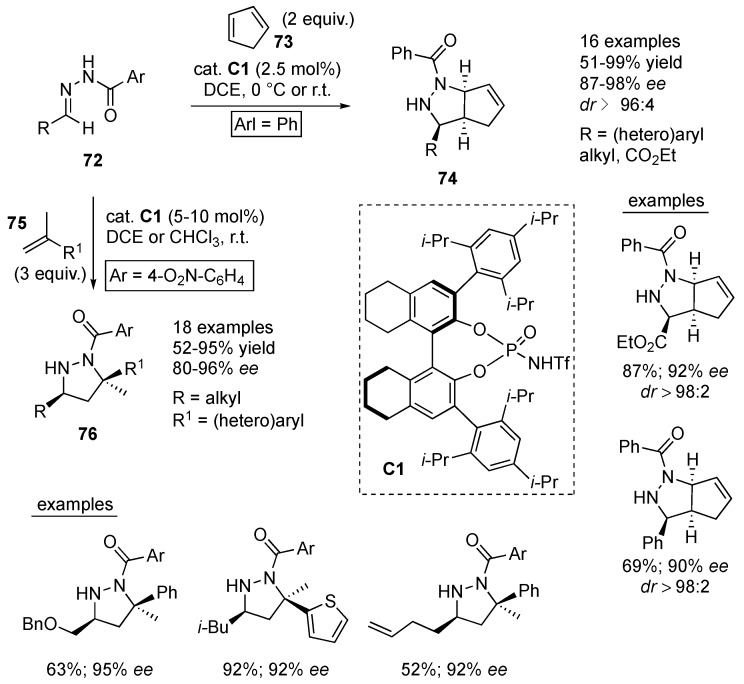
[3+2] Cycloaddition of *N*-benzoyl protected hydrazones to cyclopentadiene and *α*-methylstyrene derivatives. DCE = 1,2-dichloroethane.

Rueping et al. efficiently employed acidic *N*-triflylphosphoramide Brønsted acid **C1** as an organocatalyst in highly enantioselective cycloadditions between various alkenes and azomethine imines derived from *N*-benzoylhydrazone precursors. Thus, under optimized reaction conditions, [3+2] cycloaddition of *N*-benzoyl protected hydrazones **72** (derived from aliphatic, aromatic and heteroaromatic aldehydes or ethyl glyoxylate) to cyclopentadiene (**73**) afforded pyrazolidine derivatives **74** in 51–99% yields with excellent stereoselectivity (*dr* ≥ 96:4, 87–98% *ee*). Next, *α*-methylstyrene **75** and its (hetero)aryl analogues were used as dipolarophiles in [3+2] cycloaddition with *N*-benzoyl-protected hydrazones **72**, as azomethine imine precursors, furnishing valuable pyrazolidine derivatives **76**, bearing a quaternary and a trisubstituted stereocenter at the 3- and 5-positions, in 52–95% yields and 80–96% *ee* as single diastereomers (Scheme 24) [45].

**Scheme 25:**
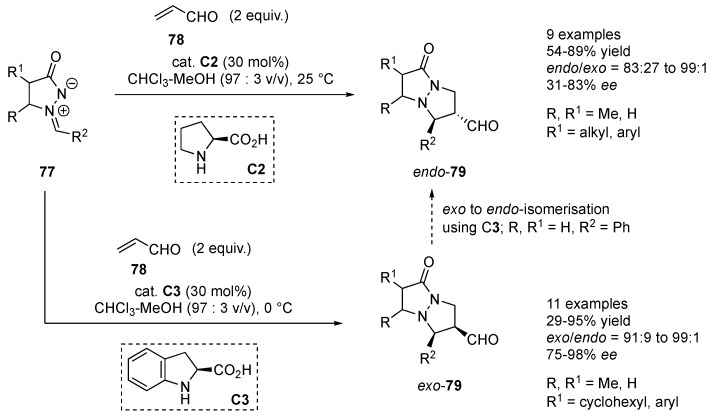
L-proline **C2**- and (*S*)-indoline-2-carboxylic acid **C3**-catalyzed cycloadditions of *N*,*N*-cyclic azomethine imines to acrolein.

Suga and co-workers reported cycloadditions of *N*,*N*-cyclic azomethine imines **77** to acrolein (**78**) catalyzed by L-proline **C2** and (*S*)-indoline-2-carboxylic acid **C3**. The initial cycloadducts **8** were, after reduction with NaBH_4_, isolated as the corresponding alcohols. Under the optimized reaction conditions, the L-proline **C2**-catalyzed reactions gave the cycloadducts **79** in 54–89% yields as the major *endo*-diastereomers (*endo*/*exo* = 83:27 to 99:1) with modest to good enantioselectivity (31–83% *ee*). Azomethine imines bearing *o*-chlorophenyl- and alkyl-substituents gave the corresponding products **79** in merely low to modest enantioselectivity (31–50% *ee*). Interestingly, reactions catalyzed by (*S*)-indoline-2-carboxylic acid **C3** furnished the corresponding cycloadducts **79** with high *exo*-selectivity (*exo*/*endo* = 91:9 to 99:1) and good to excellent enantioselectivity (75–98% *ee*), in 29–95% yields. Different substituents on the azomethine imines were well tolerated. The authors have shown that *exo*-cycloadduct can be isomerized into *endo*-cycloadduct in the presence of L-proline **C2** (Scheme 25) [46].

**Scheme 26:**
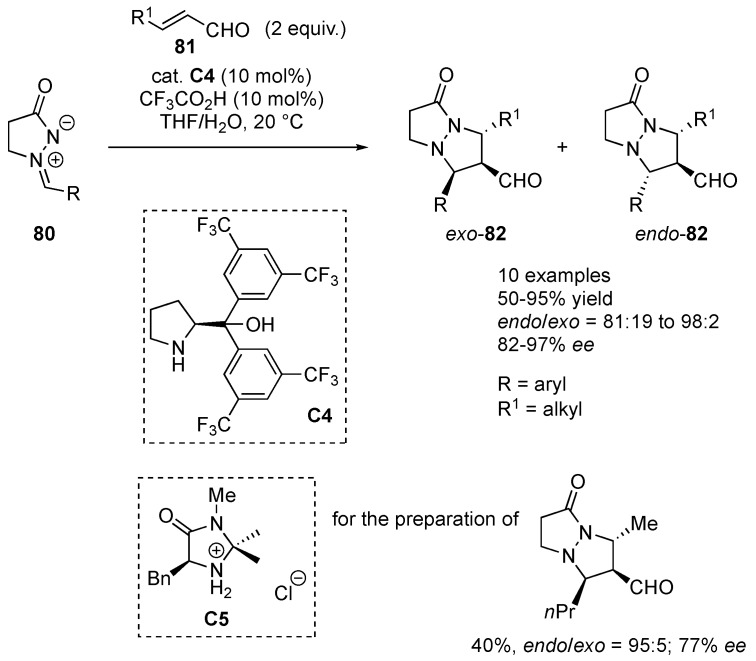
Cycloaddition of *N*,*N*-cyclic azomethine imines to α,β-unsaturated aldehydes.

α,α-Bis[3,5-di(trifluoromethyl)phenyl]prolinol **C4** was applied as the organocatalyst of choice for the stereoselective cycloaddition of *N*,*N*-cyclic azomethine imines **80** to α,β-unsaturated aldehydes **81**, furnishing the expected cycloadducts **82** in 50–95% yields with excellent stereoselectivities (*exo*/*endo* = 81:19 to 98:2; 82–97% *ee*). Both electron-withdrawing and electron-donating substituents in the *para*-position of benzene ring of azomethine imines gave the corresponding products with excellent stereoselectivities. Regarding the applied enals **81**, the ones with an alkyl group worked very well, whereas reactions with cinnamaldehyde failed to give the expected products. Azomethine imine, derived from isobutyraldehyde, was successfully reacted with crotonaldehyde in the presence of MacMillan’s first generation organocatalyst **C5** [47], giving the corresponding cycloadduct in 40% yield, 77% *ee* and *exo*/*endo* = 95:5 (Scheme 26) [48].

**Scheme 27:**
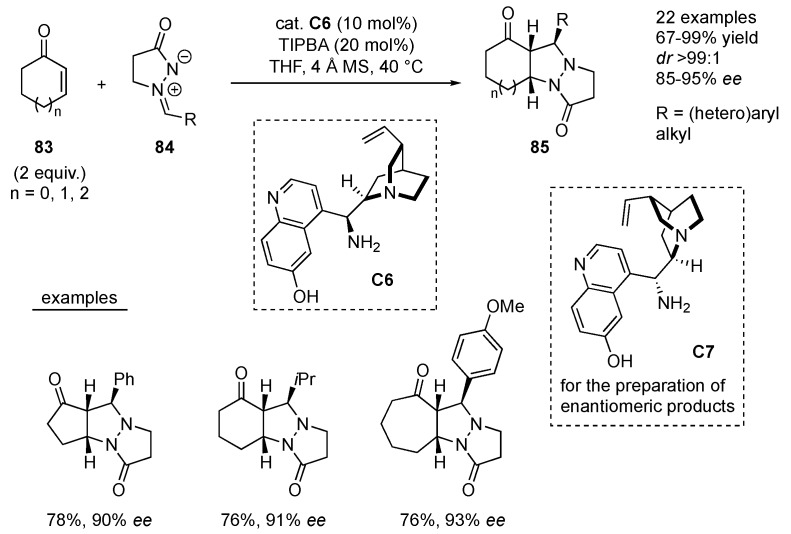
Cycloaddition of *N*,*N*-cyclic azomethine imines to 2-cyclopenten-, 2-cyclohexen-, and 2-cyclohepten-1-ones. TIPBA, 2,4,6-triisopropylbenzenesulfonic acid.

Chen et al. applied 9-amino-9-deoxyepiquinine **C6** in the presence of 2,4,6-triisopropylbenzenesulfonic acid (TIPBA) for an efficient cycloaddition of various alkyl and (hetero)aryl substituted *N*,*N*-cyclic azomethine imines **84** to 2-cyclopenten-, 2-cyclohexen- and 2-cyclohepten-1-ones **83**, giving the desired products **85** in excellent diastereoselectivity (*dr* > 99:1), excellent enantioselectivity (85–95% *ee*) and in 67–99% yields. When 9-amino-9-deoxyepiquinidine **C7** was applied, enantiomeric cycloadducts were obtained, retaining the same level of stereoselectivity (Scheme 27). The 6-hydroxy group on the quinolone part of the organocatalysts **C6** and **C7,** as well as the presence of the sterically bulky TIPBA additive were essential for the high stereoselectivity of these transformations [49].

**Scheme 28:**
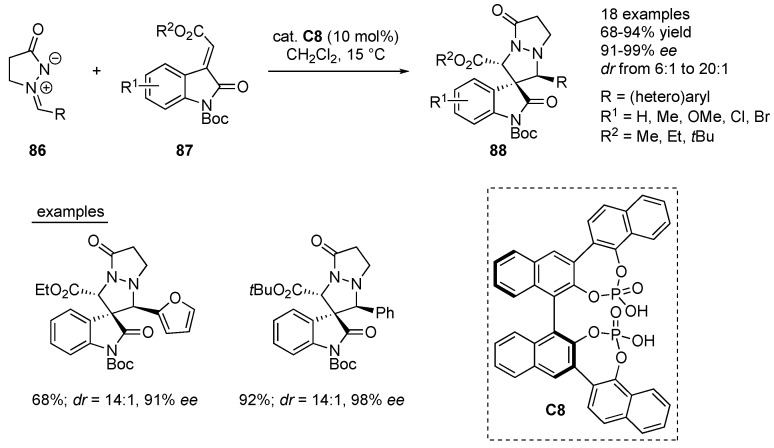
1,3-dipolar cycloaddition of *N*,*N*-cyclic azomethine imines to methyleneindolinones.

A chiral bis-phosphoric acid **C8**, bearing triple axial chirality, was developed by Wang and co-workers and successfully utilized in the 1,3-dipolar cycloaddition of *N*,*N*-cyclic azomethine imines **86** to methyleneindolinones **87**. The chiral spiro[pyrazolidin-3,3’-oxindole] products **88** have been formed in 68–94% yields, good to excellent diastereoselectivity (*dr* from 6:1–20:1) and excellent enantioselectivity (91–99% *ee*). Azomethine imines bearing different (hetero)aryl substituents, as well as different electronic nature, bulkiness or position of the substitution patterns on the methyleneindolinones had negligible effect on the efficiency and stereoselectivity of transformations under the optimized reaction conditions. DFT calculations have been performed to shed the light on the mechanism of the 1,3-dipolar cycloaddition (Scheme 28) [50].

**Scheme 29:**
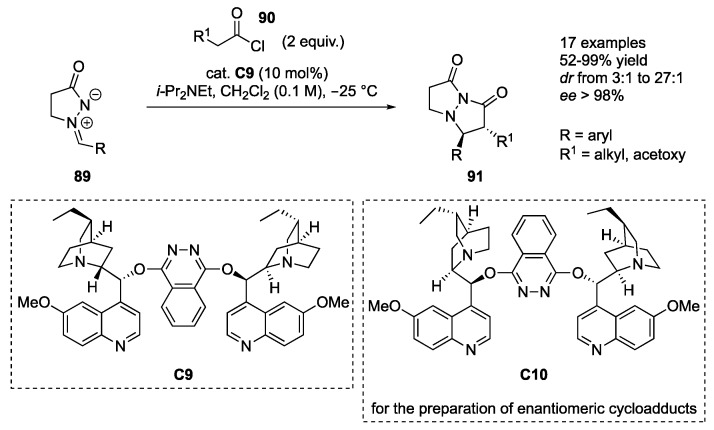
1,3-dipolar cycloadditions of ketenes to *N*,*N*-cyclic azomethine imines.

Kerrigan and co-workers reported 1,3-dipolar cycloadditions of the in situ-generated ketenes (formed from the corresponding acyl chlorides **90** and DIPEA) to azomethine imines **89** in the presence of quinuclidine organocatalyst **C9** as the catalyst of choice, thus furnishing (2*R*,3*S*)-bicyclic pyrazolidinones **91** in 52–99% yields, excellent enantioselectivity (≥96% *ee*) and moderate to high diastereoselectivity (*dr* 3:1–27:1). The method displays tolerance to aryl groups of azomethine imine containing both electron-donating and electron-withdrawing substituents, while with respect to ketene substituents, an acetoxy and different alkyl substituents were employed. With the acetoxy group, a markedly improved *trans*-diastereoselectivity was observed (*dr* 12:1 to 27:1). If pseudo enantiomeric catalyst **C10** was applied, enantiomeric (2*S*,3*R*)-bicyclic cycloadducts **91** were obtained, retaining the same level of stereoselectivity (Scheme 29) [51].

**Scheme 30:**
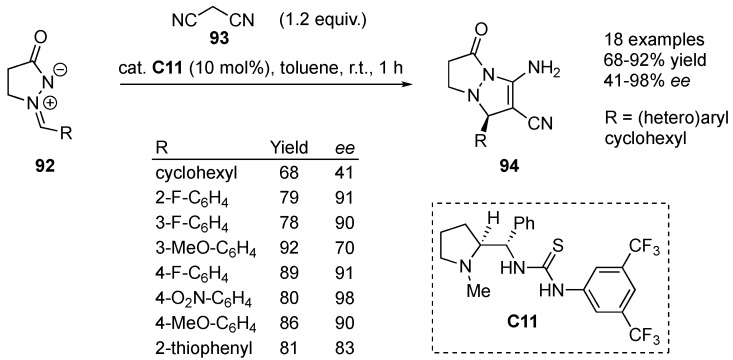
[3+2] cycloaddition of *N*,*N*-cyclic azomethine imines to malononitrile.

Proline-derived thiourea organocatalyst **C11** was used by Kesavan et al. for the formal [3+2] cycloaddition of azomethine imines **92** to malononitrile (**93**), yielding bicyclic pyrazolidinones **94** with embedded enaminonitrile functionality in 68–92% yield and 41–98% *ee*. Azomethine imines containing cyclohexyl, heteroaromatic and diversely substituted aromatic substituents were used. 2-methylmalononitrile also gave the expected product (78% yield, 70% *ee*), while other dipolarophiles such as ethyl 2-cyanoacetate, benzoylacetonitrile and (phenylsulfonyl)acetonitrile failed to react. Presumably, the azomethine imine gets activated via hydrogen bonding with the thiourea moiety, whereas the tertiary amine deprotonates/activates the malononitrile (Scheme 30) [52].

**Scheme 31:**
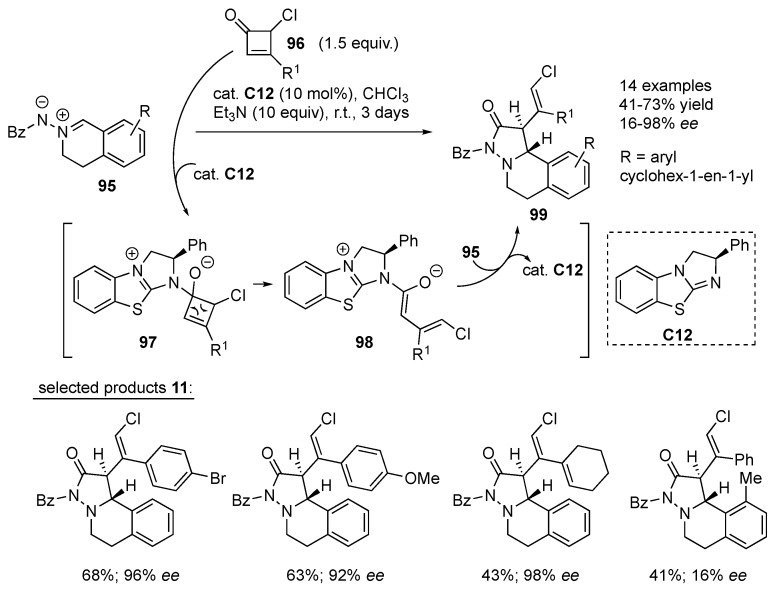
Application of *γ*-mono-chloro substituted cyclobutenones in formal [3+2] cycloadditions to *C*,*N*-cyclic azomethine imines.

Chi and co-workers developed a new C–C bond activation of *γ*-mono-chlorine-substituted cyclobutenones **96** enabled by an isothiourea Lewis base organocatalyst **C12**. The 1,2-addition of Lewis base **C12** to **96** furnishes intermediate **97**, which undergoes electrocyclic ring opening to generate vinyl enolate intermediate **98**. Selective α-addition of **98** to azomethine imine **95** followed by cyclization gives the final products **28** in excellent diastereo- and enantioselectivity (*cis*-selective, up to ≥ 98% *ee*). The developed reaction tolerates different aromatic substituents at the β-carbon of cyclobutenone **96**, while the mono-chlorine in the 3-position is essential for the positive outcome of the reaction. With regard to azomethine imine **95**, substituents in position 8 other than hydrogen result in lower yields and enantiomeric excess, presumably due to steric hindrance (Scheme 31) [53].

**Scheme 32:**
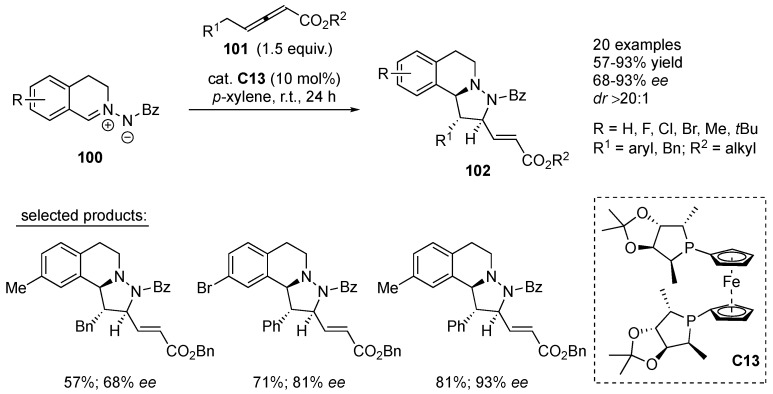
Chiral phosphine-catalyzed cycloaddition of δ-substituted allenic esters to *C*,*N*-cyclic azomethine imines.

Using chiral phosphine organocatalyst **C13**, Shi et al. managed to employ *δ*-substituted allenic esters **101** in an asymmetric [3+2] cycloaddition to *C*,*N*-cyclic azomethine imines **100**, yielding tetrahydroquinoline derivatives **102** in 57–93% yields, with high diastereoselectivity (> 20:1 *dr*) and good enantioselectivity (68–93% *ee*). The employed allenoates **101** react as C_2_-synthons via their δ- and γ-positions, for which the authors proposed a plausible reaction mechanism. Both simple and sterically-demanding alkyl allenoates **101** have been successfully employed, containing benzyl and various aryl substituents in the δ-position, while the reaction with allenoate containing *δ*-methyl-substituent failed. In the reaction of δ-Ph substituted allenoates **101** with various azomethine imines **100**, the electron-rich alkyl-substituents on the azomethine imine seem to have a beneficial influence on the enantioselectivity of the reaction. *N*,*N*-cyclic azomethine imine does not react with δ-substituted allenic esters under the applied reaction conditions (Scheme 32) [54].

**Scheme 33:**
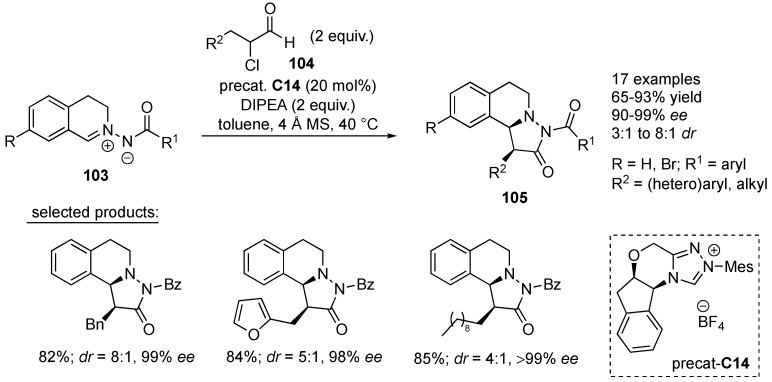
*N*-Heterocyclic carbene-catalyzed [3+2] cycloaddition of α-chloroaldehydes to *C*,*N*-cyclic azomethine imines.

Ye and co-workers developed *N*-heterocyclic carbene-catalyzed [3+2] cycloaddition of α-chloroaldehydes **104** to *C*,*N*-cyclic azomethine imines **103**. *N*-heterocyclic carbene was generated in situ from the corresponding triazolium salt **C14** and DIPEA. The corresponding pyrazolidinone products **105** were obtained in good yields (65–93%), with moderate to good diastereoselectivity (3:1–8:1 *dr*) and excellent enantioselectivity (90–99% *ee*). It was found that both β-(hetero)aryl and alkyl α-chloroaldehydes **104**, as well as azomethine imines **103** containing various *N*-arylcarbonyl groups worked well, affording the corresponding products **105** with high enantioselectivities. On the other hand, the reaction of α-chlorophenylacetaldehyde with azomethine imine, under optimized reaction conditions, furnished the desired product in decreased yield (30%) and enantioselectivity (55% *ee*), though significantly improved diastereoselectivity (*dr* > 20:1) (Scheme 33). A plausible catalytic cycle was postulated. It includes *N*-heterocyclic carbene addition to α-chloroacetaldehyde, thus generating Breslow intermediate [55], which decomposes to azolium enolate. Azolium enolate reacts with the azomethine imine in an addition-cyclization sequence, affording the final products **105** [56].

**Scheme 34:**
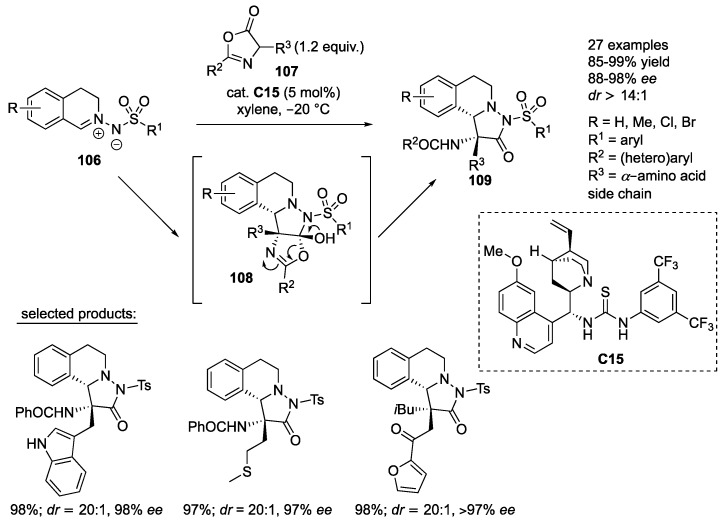
Inverse electron demand 1,3-dipolar cycloaddition of *C*,*N*-cyclic azomethine imines to azlactones.

An organocatalyzed inverse electron demand 1,3-dipolar cycloaddition of *C*,*N*-cyclic azomethine imines **106** with azlactones **107** has been established by Su and co-workers employing noncovalent quinidine-derived bifunctional thiourea organocatalyst **C15**. The initial tetracyclic intermediates **108** are unstable and rearrange into the final products **109**. Products **109** were obtained in high yields (85–99%) with excellent stereoselectivity (88–98% *ee*, *dr* ≥ 14:1). In general, azlactones prepared from different amino acids were very well tolerated, as well as azlactones containing diverse (hetero)aryl substituents in the C2-position. Azomethine imines with different substituents on the aromatic ring and various substituents at the 4-position of the phenyl ring of the arylsulfonyl protecting group were very well compatible with the developed protocol (Scheme 34) [57].

**Scheme 35:**
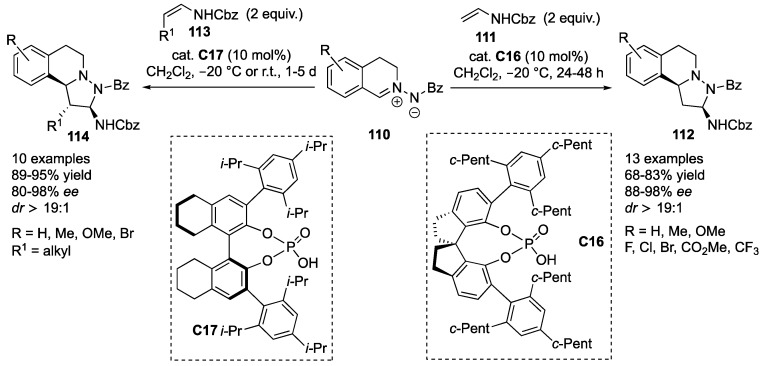
Inverse electron demand cycloaddition between *C*,*N*-cyclic azomethine imines and electron-rich enecarbamates.

Zhu et al. developed an inverse electron demand cycloaddition between *C*,*N*-cyclic azomethine imines and electron-rich enecarbamates applying chiral phosphoric acids as organocatalysts. For the cycloaddition of benzyl *N*-vinyl carbamate (**111**) to azomethine imines **110** (containing both electron-donating and electron-withdrawing substituents, irrespective of their position on the aromatic ring), phosphoric acid **C16** turned out to be the optimal catalyst, yielding the corresponding products **112** in good to high yields (68–83%) with excellent regio- and stereo-selectivity (*dr* ≥ 19:1, 88–98% *ee*). On the other hand, cycloadditions to dipolarophiles **113** possessing an electron-rich (*Z*)-internal bond needed re-optimization. Thus, the binol-based phosphoric acid **C17** was found to furnish the desired cycloadducts **114** in high yields (89–95%), excellent diastereoselectivity (*dr* ≥ 19:1) and good to excellent enantioselectivity (80–98% *ee*). Again, different substitution patterns (substituents) on the aromatic ring of azomethine imines **110** were well tolerated, whereas increasing the size of the β-substituent of the (*Z*)-enecarbamate resulted in deceleration of the reaction, which at room temperature led to decreased enantioselectivity. Finally, reaction with benzyl [(*E*)-prop-1-en-1-yl]carbamate afforded the corresponding product with significantly reduced stereoselectivity (Scheme 35) [58].

**Scheme 36:**
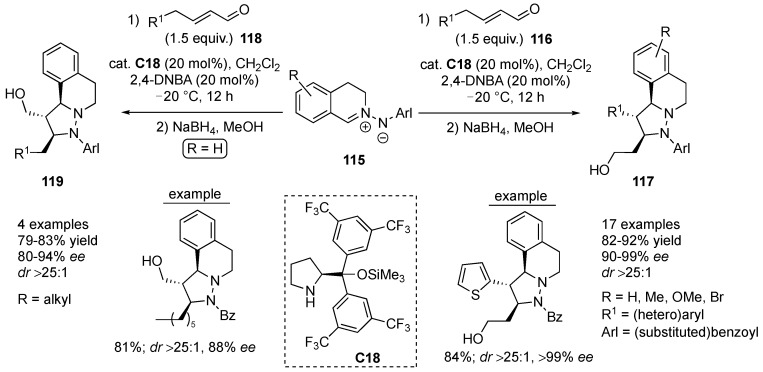
[3+2] Cycloaddition of α,β-unsaturated aldehydes to *C*,*N*-cyclic azomethine imines, controlled by iminium catalysis.

Wang and co-workers developed an asymmetric synthesis of tetrahydroquinoline derivatives via a [3+2] cycloaddition controlled by dienamine catalysis. Thus, the reaction of α,β-unsaturated aldehydes **116**, containing (hetero)aromatic substituents in the γ-position, with *C*,*N*-cyclic azomethine imines **115** (either unsubstituted or containing 5-, 6- and 7-Me/MeO/Br substituents) catalyzed by chiral prolinol silyl ether **C18** [59] furnished the desired tricyclic systems **117** in high yield (82–92%) with excellent stereoselectivity (*dr* > 25:1, 90–99% *ee*). This reaction proceeds via 4,5-reactivity of dienamine reactive intermediates. In stark contrast, reactions performed with aliphatic α,β-unsaturated aldehydes **116** with azomethine imines **115**, performed under identical reaction conditions, yielded different [3+2]-cycloaddition products **119**. In the latter case, the reaction proceeds via 3,4-reactivity of the iminium ion reactive intermediates. Products **119** were formed in 79–83% yields, *dr* > 25:1 and 80–94% *ee* (Scheme 36) [60].

**Scheme 37:**
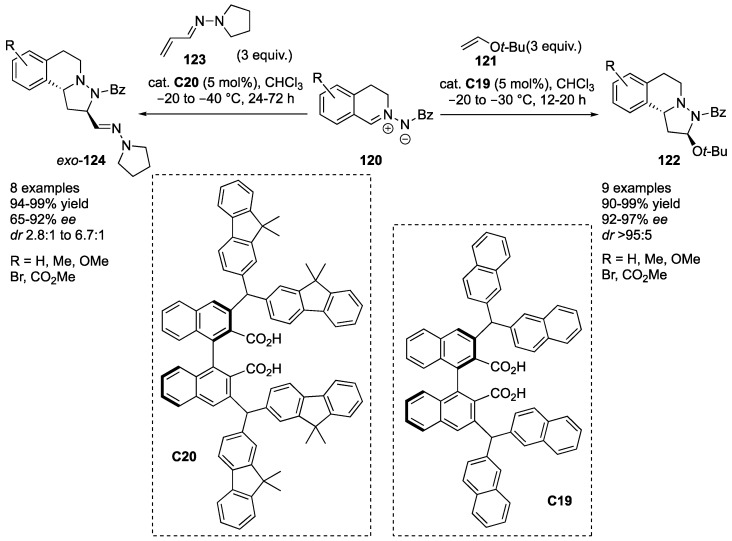
Organocatalyzed inverse electron demand cycloaddition of *C*,*N*-cyclic azomethine imines to electron-rich vinyl ether and vinylogous hydrazone.

Maruoka and co-workers developed an organocatalyzed inverse electron demand cycloaddition of *C*,*N*-cyclic azomethine imines to electron-rich vinyl ether and vinylogous hydrazone catalyzed by axially chiral dicarboxylic acids. Thus, under optimized reaction conditions (organocatalyst **C19**), azomethine imines **120**, bearing either electron-rich or electron-deficient substituents, reacted with *tert*-butyl vinyl ether (**121**) to furnish the expected cycloadducts **122** in excellent yields (90–99%) and stereoselectivities (92–97% *ee*, *exo*/*endo* > 95:5). Reactions of azomethine imines **120** with acrolein-derived vinylogous hydrazone **123** needed reoptimization of the reaction conditions (organocatalyst **C20**). The corresponding cycloadducts **124** were formed in excellent yields (94–99%) and high enantioselectivity (65–92% *ee*), albeit with modest diastereoselectivity (*exo*/*endo* = 2.8:1–6.7:1). The reaction with methacrolein-derived *α*-substituted vinylogous hydrazone generated preferentially the *endo*-diastereomer (*exo*/*endo* = 1.0:2.4) with moderate enantioselectivity (68% *ee*), while β-substituted hydrazone derived from crotonaldehyde failed to give the desired product (Scheme 37) [61].

## 4. Conclusions and Outlook

Since the seminal papers of Kobayashi [29] and Fu [13] over 30 asymmetric [3+2] cycloadditions of azomethine imines have been reported. Asymmetric cycloadditions to alkynes are limited to terminal acetylenes and the use of chiral Cu(I)-based catalysts [11]. The regioselectivity of cycloadditions is invertible by the use of Ag(I) catalysts. Although applicability of Cu(II)-based catalyst has been demonstrated [24], asymmetric cycloadditions to internal alkynes are still very scarce. Therefore, the development of asymmetric catalysts that would enable the enantioselective and regioselective preparation of cycloadducts from non-symmetrical acetylenes represents a synthetic challenge to be met. This is of particular importance, because such asymmetric reactions would allow for the preparation of analogues of Eli-Lilly’s γ-lactam antibiotics. On the other hand, the majority of catalytic asymmetric [3+2] cycloadditions of azomethine imines to alkenes have been performed using various organocatalysts, although examples of transition-metal-catalyzed reactions have also been reported [11,37,38,57]. The use of other dipolarophiles, such as cumulenes, imines, thiones, and nitriles, is another unexplored field of asymmetric cycloadditions of azomethine imines. So far, only reactions with allenoates [62], ketenes [51] and nitriles [52] have been reported.

In conclusion, the number of representative examples of asymmetric [3+2] cycloadditions of azometnine imines to various types of dipolarophiles is currently sufficient to support viability and broad scope of these reactions. However, as this research topic is now at the end if its infancy period it offers interesting and attractive challenges to synthetic chemists.
